# Laser-Guided, Self-Confined Graphitization for High-Conductivity Embedded Electronics

**DOI:** 10.34133/research.0305

**Published:** 2024-02-12

**Authors:** Haiyang Yu, Jing Bian, Furong Chen, Kan Li, YongAn Huang

**Affiliations:** ^1^ State Key Laboratory of Intelligent Manufacturing Equipment and Technology, Huazhong University of Science and Technology, Wuhan 430074, China.; ^2^ Flexible Electronics Research Center, Huazhong University of Science and Technology, Wuhan 430074, China.; ^3^ College of Electronic and Optical Engineering & College of Flexible Electronic (Future Technology), Nanjing University of Posts and Telecommunications, Nanjing 210023, China.

## Abstract

Facile fabrication of highly conductive and self-encapsulated graphene electronics is in urgent demand for carbon-based integrated circuits, field effect transistors, optoelectronic devices, and flexible sensors. The current fabrication of these electronic devices is mainly based on layer-by-layer techniques (separate circuit preparation and encapsulation procedures), which show multistep fabrication procedures, complicated renovation/repair procedures, and poor electrical property due to graphene oxidation and exfoliation. Here, we propose a laser-guided interfacial writing (LaserIW) technique based on self-confined, nickel-catalyzed graphitization to directly fabricate highly conductive, embedded graphene electronics inside multilayer structures. The doped nickel is used to induce chain carbonization, which firstly enhances the photothermal effect to increase the confined temperature for initial carbonization, and the generated carbon further increases the light-absorption capacity to fabricate high-quality graphene. Meanwhile, the nickel atoms contribute to the accelerated connection of carbon atoms. This interfacial carbonization inherently avoids the exfoliation and oxidation of the as-formed graphene, resulting in an 8-fold improvement in electrical conductivity (~20,000 S/m at 7,958 W/cm^2^ and 2 mm/s for 20% nickel content). The LaserIW technique shows excellent stability and reproducibility, with ±2.5% variations in the same batch and ±2% variations in different batches. Component-level wireless light sensors and flexible strain sensors exhibit excellent sensitivity (665 kHz/(W/cm^2^) for passive wireless light sensors) and self-encapsulation (<1% variations in terms of waterproof, antifriction, and antithermal shock). Additionally, the LaserIW technique allows for one-step renovation of in-service electronics and nondestructive repair of damaged circuits without the need to disassemble encapsulation layers. This technique reverses the layer-by-layer processing mode and provides a powerful manufacturing tool for the fabrication, modification, and repair of multilayer, multifunctional embedded electronics, especially demonstrating the immense potential for in-space manufacturing.

## Introduction

Graphene, which consists of hexagonal carbon atoms, has revolutionized the field of electronics such as integrated circuits [1–3], field effect transistors [4,5], optoelectronic devices [6,7], and flexible sensors [8,9]. Superior electrical and encapsulation properties are necessary for these graphene electronics to perform well in demanding and challenging operational conditions [10]. Currently, the layer-by-layer fabrication technique is widely used to fabricate these electronics with multilayer structures (e.g., substrate, functional circuits, and encapsulation layers), which is helpful in shielding their functional circuits from oxidation and corrosion [11–13]. This technique involves separate graphene circuit fabrication and encapsulation processes in a cleanroom [14], thus resulting in multistep, high-cost processes for the fabrication of graphene electronics and complicated procedures (disassembly, circuit preparation, and re-encapsulation) for the renovation of in-service graphene electronics or repair of the damaged graphene electronics. Among graphene circuit fabrication techniques, graphene oxide modification and laser-induced carbonization are considered 2 promising methods due to their large-scale and cost-effective potential [15]. For graphene oxide modification, the massive chemical agents and poor electrical conductivity limit its further application [16]. The laser-induced carbonization technique has been recently developed to convert the polymer into laser-induced graphene (LIG) in a high-precision, high-efficient, and pollution-free manner [17], which has been widely used in the actuators [18], electromagnetic devices [19], sensors for life sciences [20], and energy storage devices [21]. Various carbon-containing polymers, such as paper, nanocellulose, textile [22], wood, food [23], and phenolic resin [24], have been proven to be effective in the fabrication of LIG. Especially, the LIG property (e.g., electrical property) can be easily adjusted by altering the laser power or scanning speed, i.e, high laser power or low scanning speed is helpful to the sufficient carbonization, thus improving the electrical property of LIG (~ 2,500 S/m) [17]. Existing LIG processing techniques primarily focus on surface irradiation, thereby requiring additional encapsulation procedures after LIG fabrication. Unfortunately, this process often causes damage to the LIG due to its inherently porous and delicate features [25]. More importantly, this surface lasing process leads to the inevitable exfoliation and oxidation of the as-formed graphene, negatively impacting the integrity and electrical property of LIG [26,27]. These limitations hinder their further application in fields, such as wireless resonant devices and panel conductive wires. To fully exploit the potential of this scalable approach, there is a need to develop fabrication techniques that prevent the exfoliation and oxidation of the graphene and improve its electrical property. Simultaneously, it is crucial to integrate the fabrication and encapsulation processes into a single step to minimize the potential damage to LIG.

In this work, we propose an interfacial processing technique, referred to as laser-guided interfacial writing (LaserIW), which is a 1-step strategy for the direct fabrication of highly conductive graphene circuits inside multilayer structures, without the need for a cleanroom and additional encapsulation processes. Highly conductive embedded circuits are achieved via nickel-catalyzed interfacial carbonization reactions, with ~8 times enhancement in the electrical conductivity of LIG. The embedded graphene circuits are generated in confined and oxygen-free environments, which prevents the exfoliation and oxidation of the as-formed graphene that inevitably occurs with conventional surface laser-induced carbonization techniques. Besides, the limited heat-affected depth to the lower layer allows for the self-encapsulation of graphene circuits, and their functions and performances can be accurately controlled in a digital manner. Various multilayer structures are utilized to demonstrate the multiple application prospects of direct-written embedded graphene circuits, including passive wireless sensors for light detection and flexible strain sensors for gesture recognition. Furthermore, the LaserIW technique allows for the direct modification/repair of in-service multilayer electronics (e.g., adjusting the amplification factor of amplifier circuits, adding new functions, and repairing damaged circuits on panels), without the need to disassemble encapsulation layers. These collective results exhibit the promising prospects of the LaserIW technique for fabricating, modifying, or repairing graphene electronics, providing a new avenue for in-space manufacturing.

## Results

### Preparation of multilayer embedded electronics by the LaserIW technique

**Fig. 1.**
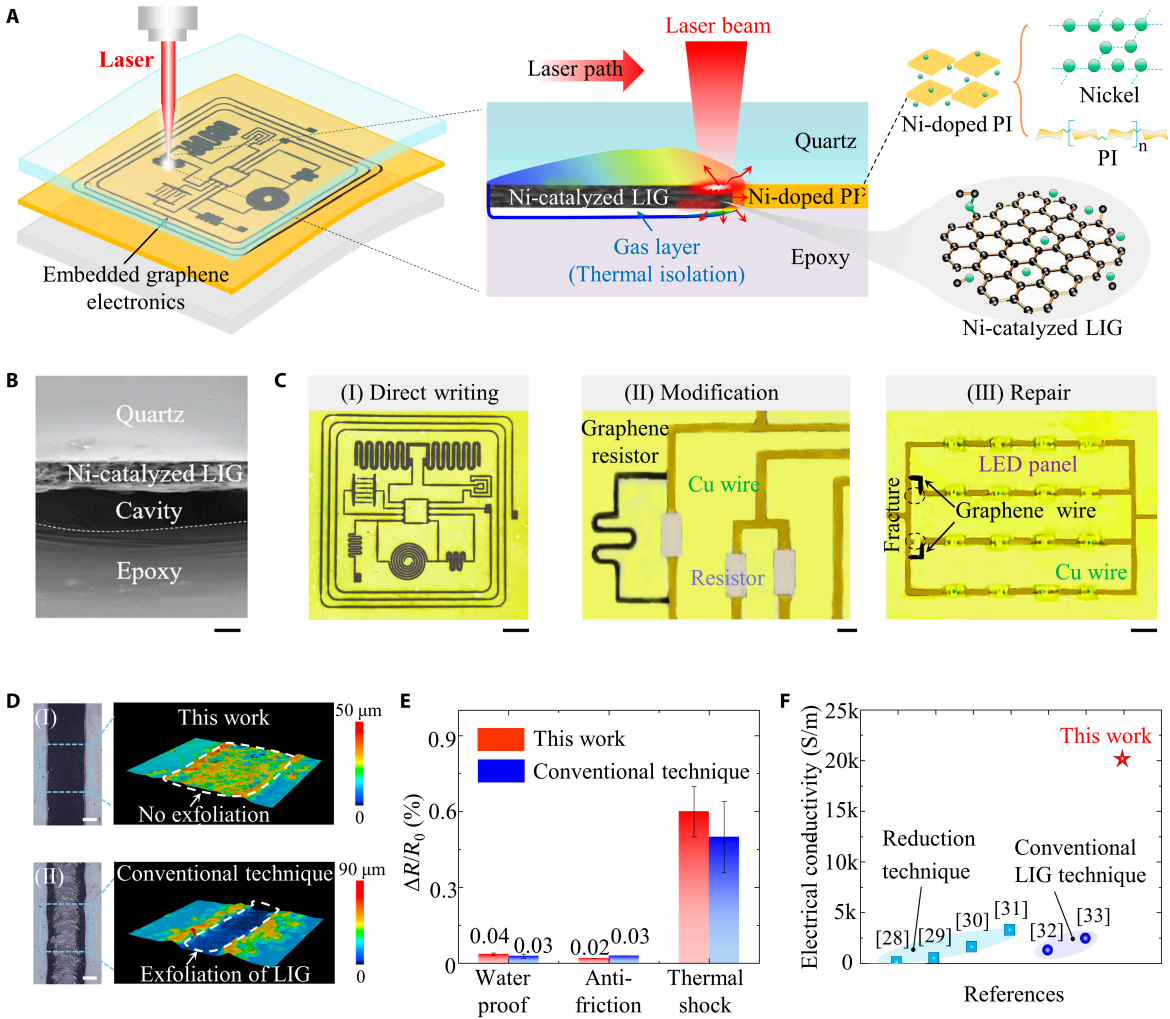
Design of LaserIW technique for multilayer embedded graphene electronics. (A) Schematic diagram of the LaserIW technique for the fabrication of embedded graphene electronics. (B) Cross-section (scale bar 40 μm) morphology at 3183 W/cm^2^ and 2 mm/s. (C) Three functions of the LaserIW technique include (I) direct-writing (scale bar 6 mm) (II) modification (scale bar 1 mm), and (III) repair (scale bar 1 mm). (D) Optical images and height cloud maps of LaserIW and conventional surface laser-induced carbonization at 3183 W/cm^2^ and 2 mm/s (scale bar 200 μm). (E) Encapsulation performance tests of the samples prepared by LaserIW and conventional techniques. (F) Comparison of the electrical conductivity of LIG prepared by LaserIW and existing methods.

Figure 1A exhibits the schematic diagram for the fabrication of multilayer embedded graphene electronics by the LaserIW technique. Here, an 808-nm infrared laser is selected as the excitation source to prepare the embedded circuits inside a pre-prepared multilayer structure (see the detailed preparation process in Fig. S1). In this structure, the quartz glass is transparent to the infrared laser while interfacial nickel-doped polyimide is highly absorptive to this infrared laser (Fig. S2), which allows the laser to penetrate through the top quartz glass to carbonize the interfacial nickel-doped polyimide into nickel-catalyzed LIG. The cross-section morphology (Fig. 1B) shows that nickel-catalyzed LIG is tightly connected with quartz glass, and a cavity is formed between the nickel-catalyzed LIG and the epoxy, which prevents further destructive ablation of the underlying epoxy. In addition, the LaserIW technique is also compatible with other multilayer structures to fabricate flexible embedded electronics, such as the PZT/nickel-doped polyimide/commercial VHB structure in Fig. S3A.

The proposed LaserIW technique enables (a) direct writing of embedded graphene electronics, (b) performance modification of original encapsulated circuits in one step, and (c) nondestructive repair of damaged circuits (no requirement to disassemble encapsulation layers). As seen in Fig. 1C (I), the graphene wires are used to connect the microcontroller with different electronic components to fabricate a state-of-the-art embedded electronic system. Other complicated patterns can be also directly patterned inside multilayer structures without a mask, such as the logo of Huazhong University of Science and Technology in Fig. S3C. Beyond these fundamental capabilities, the embedded graphene resistor is fabricated and connected in parallel with the resistor of in-service electronic devices to tune its performance (Fig. 1C [II]). In addition, the LaserIW technique can prepare an embedded graphene wire to enable the electrical reconnection of damaged copper wires without the need to disassemble encapsulation layers, as exhibited in Fig. 1C (III).

Comparing conventional surface processing techniques, the LaserIW technique shows 3 unique advantages. Firstly, the as-formed LIG is confined inside multilayer structures to avoid its exfoliation and oxidation that inevitably occurs in the conventional surface laser-induced carbonization (Fig. 1D). Secondly, the electronics fabricated by the LaserIW technique exhibits self-encapsulation (no additional encapsulation procedures). In particular, the encapsulation performance is comparable to conventional techniques (LIG samples are encapsulated by additional procedures) in terms of water proof, antifriction, and thermal shock (Fig. 1E). Finally, the graphene fabricated by the LaserIW technique shows the highest electrical conductivity of ~ 20,000 S/m [28–33], as exhibited in Fig. 1F and Table S1.

### The effect of nickel on LIG formation

**Fig. 2.**
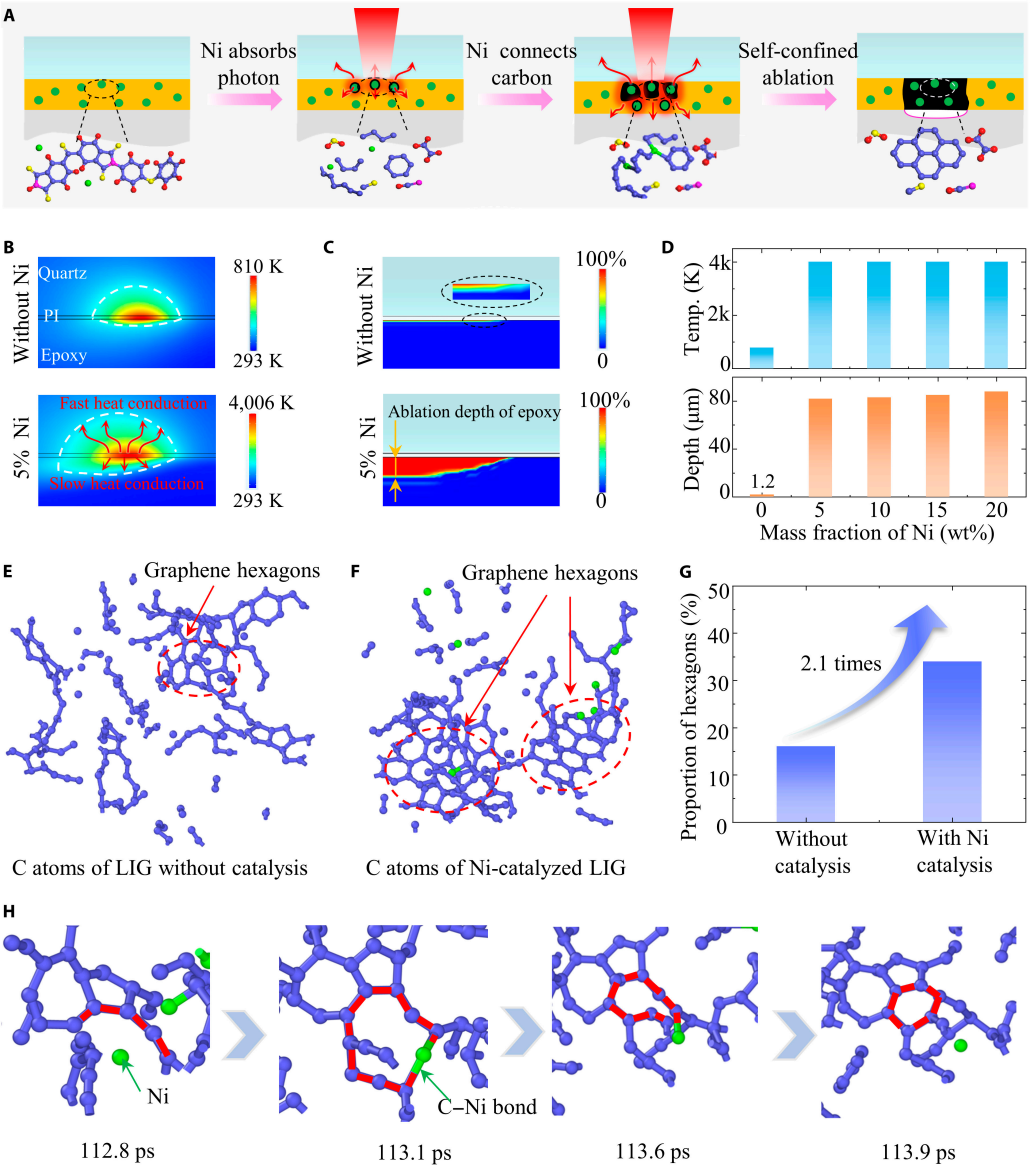
Mechanism of nickel effect on graphene formation. (A) Interfacial graphitization process of nickel-doped polyimide during laser irradiation. (B) Temperature field distributions of pure polyimide and nickel-doped polyimide (5% nickel content) at 3183 W/cm^2^ and 2 mm/s. (C) Ablation depths of epoxy for pure polyimide and nickel-doped polyimide (5% nickel content) at 3183 W/cm^2^ and 2 mm/s. (D) Relationship of nickel content on the maximum temperature of interfacial polyimide and ablation depth of epoxy at 3183 W/cm^2^ and 2 mm/s. (E) Distribution of carbon atoms of pure polyimide at 400 ps. (F) Distribution of carbon atoms of nickel-doped polyimide at 400 ps. (G) Proportion of hexagons with and without nickel catalysis. (H) Formation process of graphene hexagons under nickel catalysis.

Figure 2A shows the interfacial graphitization process of nickel-doped polyimide during laser irradiation. Firstly, the doped nickel absorbs photon energy to heat up and subsequently transfers this heat to the surrounding polyimide for small-area carbonization. Then, the generated amorphous carbon will further increase the light**-**absorption capacity to enlarge carbonization areas, and the nickel connects the free carbon atoms to transform the amorphous carbon into high-quality graphene. Finally, the entire thickness of nickel-doped polyimide is transformed into conductive LIG in a confined and oxygen-free environment, and the as-formed LIG is embedded inside the multilayer structure. In the graphitization process, the first role of nickel is to enhance the laser-induced photothermal effect to increase the confined temperature. Based on the proposed photothermal model [34,35] (see the Supplementary Materials and Methods for more details), the temperature distribution and chemical decomposition are numerically obtained, as presented in Fig. 2B and C. As can be seen, the maximum temperature of nickel-doped polyimide (5% nickel content) is ~5 times higher than the pure polyimide, and the ablation depth of epoxy is ~40 times greater. As the residual solid decomposition products of epoxy are very small (<5%) [36], this ablation will form a gas-filled cavity that could act as a thermal isolation layer to avoid further damage to the underlying epoxy. This self-limited effect of interfacial carbonization on the lower layers ensures the self-encapsulation property. The maximum temperature and the ablation depth remain unchanged when further increasing the nickel content from 5% to 20% (Fig. 2D). This is due to nickel-induced chain carbonization, during which the nickel-doped polyimide could easily reach its critical carbonization temperature (873 K) to form amorphous carbon with higher light-absorbance. Meanwhile, the power density and scanning speed have effects on the maximum temperature and the ablation depth [37–39], as exhibited in Figs. S4 and S5. As the power density increases from 3,183 to 7,958 W/cm^2^, the maximum temperature of pure polyimide increases from 780 to 9,608 K at a scanning speed of 2 mm/s, accompanied by the increase in the ablation depth of epoxy from 1.2 to 175 μm. Similar trends can be found for the decreasing scanning speed, i.e., the maximum temperature increases from 3,608 to 4,557 K when the scanning speed decreases from 4 mm/s to 1 mm/s at a fixed power density of 3,183 W/cm^2^, with the increasing ablation depth of epoxy from 50 to 129 μm.

Additionally, the catalytic effect of the nickel is responsible for the improved quality of graphene. The ReaxFF molecular dynamics method is used to investigate the effect of nickel on graphene formation at the atomic scale (Fig. S6 and Movies S1 and S2). The simulation results demonstrate that the molecular chain of polyimide would break and rejoin at high temperatures to form a sheet-like carbocycle (Fig. 2E and F). The proportion of graphene hexagons formed with nickel catalysis is 2.1 times higher than that without catalysis (Fig. 2G), indicating that nickel is favorable to lower defect ratios. A short-term diagram of the formation of graphene hexagon (Fig. 2H) reflects the specific role of nickel: (a) carbon atoms generated by the decomposition of polyimide form open-ring carbon chains, (b) nickel atoms link these open-ring carbon chains into closed rings and attract the surrounding carbon atoms to form hexagons, and (c) nickel atoms move to another open-ring position to continue the catalysis process. This effect induces more carbon atoms to form carbon clusters, resulting in an increased carbon proportion in the products. These theoretical results agree well with the experiments of the thermogravimetric analysis (Fig. S7).

### Characterization of LIG

**Fig. 3.**
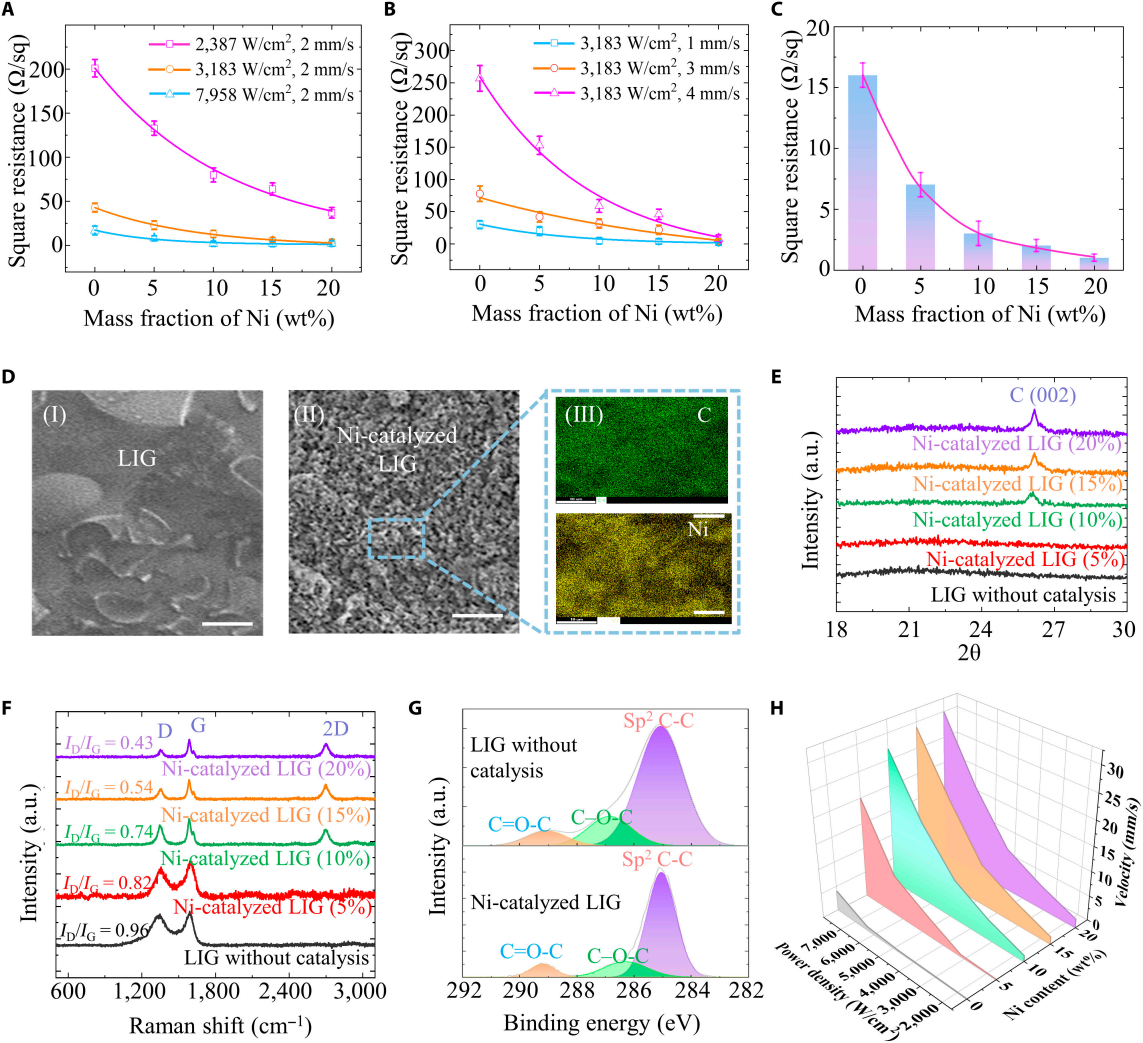
LIG characterization and performance testing. (A) Relationship between square resistance of LIG and power density and nickel content. (B) Relationship between square resistance of LIG and scanning speed and nickel content. (C) Minimum square resistance achievable with different nickel contents. (D) Characterization tests at 3183 W/cm^2^ and 2 mm/s include (I) the morphology of LIG (scale bar 10 μm), (II) the morphology of nickel-catalyzed LIG (scale bar 10 μm), and (III) a magnified view of EDS (scale bar 2 μm). (E) XRD test results of LIG with different nickel contents at 3183 W/cm^2^ and 2 mm/s. (F) Raman spectra of LIG with different nickel contents at 3183 W/cm^2^ and 2 mm/s. (G) XPS characterization results of LIG and nickel-catalyzed LIG at 3183 W/cm^2^ and 2 mm/s. (H) Relationship between graphene process window and power density, scanning speed, and nickel content.

The power density, scanning speed, and nickel content play critical roles in adjusting the electrical property of LIG. Generally, the strategy of higher power density or lower scanning speed is beneficial to improve the electrical property of LIG due to more sufficient carbonization reactions (Fig. 3A and B). Interestingly, the resistance of LIG monotonously decreases with the increase of power density or decrease of scanning speed, followed by saturation (Fig. S8). This trend is different from surface laser-induced carbonization, where the resistance will first decrease and then increase (Fig. S9). This is because the LIG formed by the surface laser-induced carbonization technique could be easily peeled off due to the large temperature stress [40,41], while the LIG is constrained to avoid its exfoliation for the LaserIW technique. This unique interface-confined feature attributes to enlarge the process parameter window for achieving a minimum square resistance of LIG. Moreover, the doped nickel helps to lower the square resistance of LIG. As seen in Fig. 3C, the minimum square resistance of LIG monotonically decreases to 1 Ω/sq with an electrical conductivity of ~20,000 S/m, as nickel content increases to 20% under the same process parameters of 7,958 W/cm^2^ and 2 mm/s. The rate of resistance reduction sharply decreases at nickel content greater than 10%, suggesting that this ratio of nickel to polyimide reaches a critical saturated value, beyond which the resistance reduction effect will be substantially weakened.

The effect of doped nickel on the morphology of carbonization products has been confirmed by SEM observations. As seen in Fig. 3D (I, II), SEM images show that the morphology of nickel-catalyzed LIG is more uniform than that of LIG (3,183 W/cm^2^ and 2 mm/s). This phenomenon also exists when increasing the power density from 3,183 to 7,958 W/cm^2^ or decreasing the scanning speed from 4 to 2 mm/s (Figs. S10 and S11). With the nickel content increased from 0 to 20%, the morphology of nickel-catalyzed LIG first becomes uniform in some limited areas (5% nickel), and then the uniform areas cover all zones and remain almost unchanged (10% to 20% nickel). After laser irradiation, the doped nickel is still uniformly distributed in the LIG (Fig. 3D [III]).

The doped nickel contributes to high-quality carbonization products. X-ray diffractometer (XRD) results show that the bond of (002) appears at 26° once the nickel content is greater than 10% at a fixed process parameter, representing an ordered layer structure with an interplanar spacing of ~0.34 nm (Fig. 3E). Raman spectroscopy is further used to accurately identify the specific species of carbonization products, such as amorphous carbon with D (~1,350 cm^-1^) and G peaks (~1,580 cm^-1^), and ordered graphene with D, G, and 2D peaks (~2,700 cm^-1^) (Fig. S12). The results show that only D and G peaks are present when the nickel content is below 10%, whereas a 2D peak emerges when the nickel content surpasses 10% (Fig. 3F). As the nickel content increases from 0 to 20%, the I_D_/I_G_ (defect ratio) decreases from 0.96 to 0.43, indicating that the doped nickel reduces the defects of carbonization products. X-ray photoelectron spectroscopy (XPS) analysis shows that the peak width of C (sp^2^) and the area of C=O-C and C-O-C of nickel-catalyzed LIG are smaller, suggesting that nickel-catalyzed graphene has fewer impurities (Fig. 3G). These more ordered and purer features endow carbonization products with better electrical property [42].

In addition, the doped nickel also has great effects on the process window for the fabrication of graphene by the LaserIW technique, which is obtained by batch experiments of carbonization product analysis. The results demonstrate that the process window of pure polyimide is very narrow as the gray area in Fig. 3H. By contrast, as the nickel content increases to 10%, a marked larger process window can be obtained, beyond which the effect of nickel on the graphene process window becomes saturated. This trend is the same as the other characterization results, demonstrating that a 10% nickel content is an optimum ratio.

### Preparation and characterization of embedded graphene electronics

**Fig. 4.**
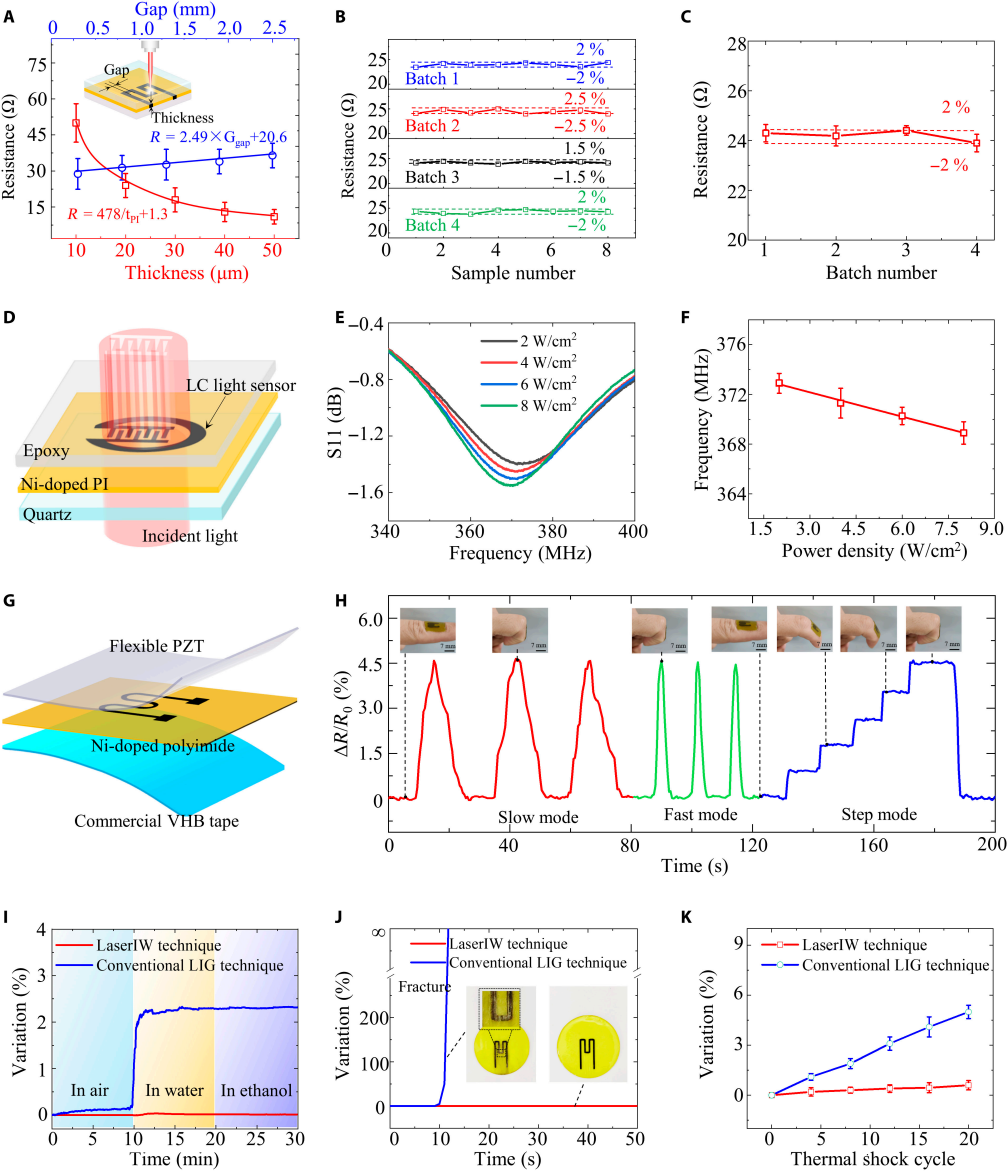
Preparation and characterization of embedded electronics fabricated by the LaserIW technique. (A) Correlations of thickness and gap with resistance. (B) Resistance variations at different locations in the same batch for 4 sample batches. (C) Resistance error from batch to batch. (D) Application background of LC light sensor for a smart window. (E) S11 parameters at different optical power densities. (F) Correlation of resonant frequency with optical power density. (G) Structural diagram of flexible strain sensors. (H) Strain sensor works for gesture recognition in 3 modes. (I) Resistance variation of devices fabricated by LaserIW and conventional surface laser-induced carbonization techniques in air, water, and ethanol environments. (J) Antifriction performance comparison of devices fabricated by the LaserIW technique and surface laser-induced carbonization. (K) Thermal shock performance comparison of devices fabricated by the LaserIW technique and surface laser-induced carbonization technique.

Different embedded electronic components, including resistors, capacitors, and inductors, could be directly fabricated inside multilayer structures (e.g., a 3-layer structure of quartz/Ni-doped polyimide/epoxy) in one step. The performance of these electronic components can be adjusted by the ablation thickness and geometric sizes, as illustrated in Fig. 4A and Fig. S13. In addition, nickel has an effect on the performance of resistors and capacitors. As seen in Fig. S14, the resistance of nickel-catalyzed LIG is ~16 times smaller than that of LIG without nickel catalysis. This excellent electrical property is beneficial to broaden the application fields of LIG, such as highly conductive wires for wireless resonant sensors. For the capacitor, the capacitance of nickel-doped LIG is 1.4 times larger than that of LIG without nickel catalysis due to the larger charge density [43].

Four batches of embedded electronics (3-layer structure of quartz/Ni-doped polyimide/epoxy) are prepared to verify the reproducibility and stability of the process and structures, with 8 resistors in each batch (Fig. S15A). As seen in Fig. 4B, the variation of the 8 resistors in the same batch but in different spatial locations is within ±2.5%, showing uniform nickel distribution and good process stability. As for the performance error from batch to batch, it varies within ±2% (Fig. 4C), demonstrating the excellent reproducibility of the LaserIW technique. In addition, the aging effect of the embedded electronics is tested, with device performance varying within ±1% after 2,000 cycles (each lasting 120 s), as illustrated in Fig. S15B.

The nickel-catalyzed LIG exhibits high absorbance at wavelengths ranging from 250 to 850 nm (Fig. S16A), while the uncarbonized region remains transparent. Based on this feature, smart windows with the ability to detect the intensity of external incident light can be fabricated (Fig. 4D). Here, a rigid multilayer structure of quartz glass/nickel-doped polyimide/epoxy is designed to prepare an LC passive wireless resonant light sensor, which consists of a light-sensitive interdigital capacitor connected with a single-turn inductor. In this design, a coil placed above the sensor is used for the inductively coupled link between the LC passive light sensor and the readout system, enabling wireless signal transmission (Fig. S16B). The resonant frequency of the passive light sensor is expressed as $f=1/2\pi \sqrt{\mathrm{LC}}$, where *L* and *C* represent the inductance and capacitance of the sensor, respectively. The resonant frequency decreases with increasing optical power density (Fig. 4E) since the photon absorption by the LIG leads to an increase in capacitance [44]. This variation can be wirelessly sensed by the upper copper coil (10-mm diameter). As illustrated in Fig. 4F, there is a good linear relationship between the resonant frequency and optical power density ranging from 2 to 8 W/cm^2^ with an average sensitivity of 665 kHz/(W/cm^2^).

In addition, the LaserIW technique is also compatible with other multilayer structures for some scenarios requiring flexible electronics. As a demonstration, a 3-layer flexible strain sensor (PZT/Ni-doped polyimide/VHB) is fabricated by the LaserIW technique, which consists of a PZT encapsulation layer (thickness of 1 μm; synthesis process in Methods) on top, a nickel-doped polyimide layer, and a commercial VHB tape on the bottom (see Fig. S17 for more details about the preparation process), as exhibited in Fig. 4G. Since PZT is transparent to 808-nm laser and highly absorbing to 308-nm laser, the flexibility of the device is achieved by first preparing the embedded strain sensor with an infrared laser and then peeling the sapphire substrate with a UV laser. As exhibited in Fig. 4H, the fabricated strain sensor works well to monitor different gestures in slow, fast, and step modes.

The encapsulation capability of the samples fabricated by the LaserIW technique, such as liquid-proof, antifriction, antithermal shock, and antitear, has been fully tested. For comparison, samples fabricated by the surface laser-induced carbonization technique without encapsulation are also tested (Figs. S18 and S19). The performance of components fabricated by the LaserIW technique remains unchanged in the environment of liquid (Fig. 4I), friction (Fig. 4J), and thermal shock (Fig. 4K), while the sample fabricated by the surface laser-induced carbonization technique shows a great variation (e.g., 2.3% resistance variation in the liquid-proof test, electrode fracture in the antifriction test, 5% resistance variation in the antithermal-shock test). Furthermore, these samples prepared by the LaserIW technique also pass the tape adhesion test. The internal graphene pattern remains intact without leaving any residue on the tape after tearing, whereas the pattern produced by the surface laser-induced carbonization technique tends to tear off (Fig. S20). The excellent self-encapsulation properties promote the versatile applications of the LaserIW technique.

### Integrated processing of the LaserIW technique for in-space manufacturing

**Fig. 5.**
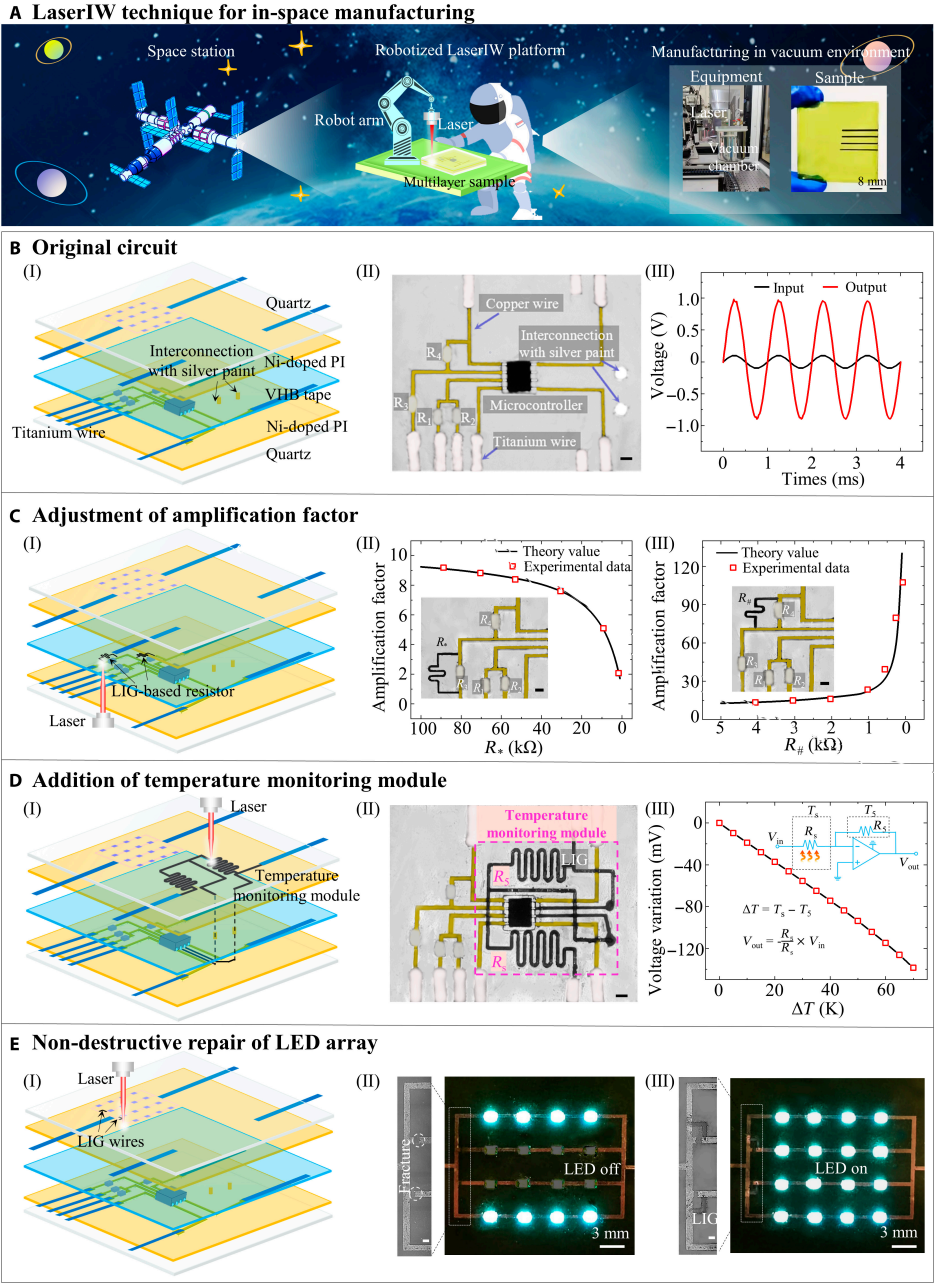
Integrated processing of LaserIW technique for in-space manufacturing. (A) Schematic diagram of the LaserIW technique applied to in-space manufacturing. (B) Original circuit includes (I) schematic diagram, (II) top view of the original circuit (scale bar of 2 mm), and (III) output and input voltage signals. (C) Adjustment of amplification factor includes (I) schematic diagram and correlation of the theoretical and experimental amplification factor with the parallel resistance of (II) R_*_ and (III) *R*_#_. The inset image is a top view of the modified circuit (scale bar of 1 mm). (D) Addition of a temperature monitoring module on another layer includes (I) schematic diagram, (II) top view of the new circuit with temperature monitoring module (scale bar of 2 mm), and (III) voltage variation at different temperature differences. (E) Nondestructive repair includes (I) schematic diagram, (II) an LED array with 2 rows of fracture circuits before repair, and (III) an LED array after repair; scale bar of 600 μm.

In-space manufacturing technology that prepares various electronics directly in space is a very forward-thinking but practical idea, which requires lightweight and compact manufacturing equipment, on-demand fabrication depending on the actual space environment, and no pollution to the space environment. Meanwhile, the ability to quickly modify the performance and functionality of these electronic devices and nondestructively repair their damaged circuits can further increase their applicability and flexibility. Conventional layer-by-layer processing involves complicated circuit preparation and encapsulation procedures, and the corresponding devices tend to be nonportable for the limited room of the space station. The LaserIW technique only requires a portable laser and a basic motion platform (or robot arm), which presents a new avenue to fabricate electronic devices, modify their performance and functionality, and nondestructively repair their damaged circuits. To mimic the vacuum environment of the space station, a vacuum chamber (-0.095 MPa) with top transparent glass is selected (Fig. 5A; see Fig. S21A for more details). As can be seen, the vacuum environment has no noticeable effect on the fabrication of electronic devices. Even at the high laser fluence of 7,957 W/cm^2^ and 1 mm/s, the embedded electronics maintain excellent structural integrity, without layer delamination (Fig. S21B). This is attributed to the excellent adhesion properties between epoxy and Ni-doped polyimide as well as the sufficient stiffness of the sample structure. With the basic fabrication abilities of the LaserIW technique characterized, we now exhibit its unique ability to directly write embedded LIG wires to renovate/repair the in-service electronic system in ways that are previously unattainable with conventional processing technologies.

As a demonstration, a multilayer amplifier circuit (5-layer structure of quartz/Ni-doped polyimide/VHB/Ni-doped polyimide/quartz in Fig. 5B [I, II]) is modified using the LaserIW technique to show its unique ability in processing circuits. The output voltage (*V*_*out*_) is related to the input voltages (*V*_*in*1_ and *V*_*in*2_) and resistance of *R*_3_ and *R*_4_, as *V*_*out*_ = *R*_3_ + *R*_4_ × (*V*_*in*1_ + *V*_*in*2_)/2*R*_3_ [45]. Initially, the output voltage is 0.98 V when supplied with two 100-mV sinusoidal voltage signals (Fig. 5B [III] and Fig. S22), which is very close to the predesigned amplification factor (*β* = *V*_*out*_/*V*_*in*_) of 10. Generally, this value is unlikely to be modified. Here, the LaserIW technique allows the preparation of embedded resistors connected in parallel with *R*_3_ and *R*_4_ on the same layer (Fig. 5C [I]), thus enabling convenient adjustment of the amplification factor. By fabricating a parallel resistor of *R*_4_, the original amplification factor decreases with the increase of resistance (Fig. 5C [II]). Conversely, the parallel resistor in the *R*_3_ branch increases the amplification factor, which increases as the parallel resistance decreases, as shown in Fig. 5C (III). This processing technique enables a large modulation range of approximately ~100-fold for the amplification factor, making it an efficient solution for adjusting electronic performance, such as the transmission distance of the radio frequency amplifier [46,47].

Additionally, the original amplifier circuit can be easily added with new functions, even if it has been already encapsulated. As a demonstration, a temperature monitoring module is added on another layer to show the unique renovation ability of the LaserIW technique for multilayer circuits (Fig. 5D [I, II]). The output voltage is proportional to the resistance ratio and the input voltage, as *V*_*out*_ =  - *R*_6_/*R*_5_ × *V*_*in*_. The resistance of LIG shows a negative correlation with temperature (Fig. S23), which causes a variation in the output voltage when 2 sensors are at different temperatures (Fig. S24A and B). As expected, the voltage variation increases as the temperature difference increases, as exhibited in Fig. 5D (III). Besides, this temperature monitoring module even has a response when a human hand is near the sensor (Fig. S24C), demonstrating its strong ability to monitor small temperature variations.

Notably, the proposed LaserIW technique is especially suitable for repairing multilayer damaged electronics. As proof of the concept, a light-emitting diode (LED) array with 2 rows of broken circuits is selected to demonstrate the nondestructive repair capability of the LaserIW technique. Specifically, the embedded LIG wires are directly prepared at the edge of the broken circuit to electrically reconnect them (Fig. 5E [I]). As seen in Fig. 5E (II, III), the 2 rows of LEDs are lighted up again. This one-step repair strategy substantially simplifies the conventional repair procedures (disassembly, circuit preparation, and re-encapsulation). The facile repair of the LaserIW technique provides a new scheme for the effective management of circuit-damaged electronics, which are usually discarded as electronic wastes.

## Discussion

In summary, we first develop an interfacial processing technique known as laser-guided interfacial writing (LaserIW) to directly fabricate highly conductive, embedded graphene circuits inside multilayer structures. The doped nickel enhances the photothermal effect and promotes the formation of the carbocycle, thus resulting in an 8-times enhancement in the electrical conductivity of LIG (~20,000 S/m at 7,958 W/cm^2^ and 2 mm/s for 20% nickel content). Sandwiched fundamental electronic components, such as resistors, capacitors, inductors, and their random combinations, can be fabricated in one step using this technique, with excellent self-encapsulated performance (<1% variations in terms of waterproof, antifriction, and antithermal shock). In addition, the LaserIW technique offers excellent stability and reproducibility, demonstrating ±2.5% difference within a batch and ±2% difference between batches. Different types of multilayer structures allow the design and fabrication of various embedded graphene electronics, such as the quartz/Nickel-doped polyimide/epoxy structure for wireless light sensors and PZT/Nickel-doped polyimide/VHB structure for flexible strain sensors. In particular, the LaserIW technique is favorable for some occasions that cannot be processed by the existing surface processing techniques, such as the renovation of in-service electronics and the nondisassembled repair of circuits inside multilayer structures (e.g., quartz/Ni-doped polyimide/VHB/Ni-doped polyimide/quartz). These advances exhibit the great potential of the LaserIW technique for the facile fabrication, modification, and nondestructive repair of embedded electronics, presenting promising applications in the field of in-space manufacturing. In addition to the innovative developments described above, there are 2 limitations to this study. First, the linewidth is greater than 100 μm, which is too wide on some occasions. This problem can be solved by using an ultrafast laser with a smaller spot radius. Second, the main focus of this study is to validate that the LaserIW technique can be used to prepare embedded graphene electronics with high conductivity, while relatively little research has been done on their applications. In the future, more attention will be paid to the preparation of high-resolution graphene wires and the exploration of potential applications.

## Materials and Methods

### Fabrication process of multilayer sample

The polyimide precursor solution is purchased from Beijing Bomi Technology Co., Ltd (China). The nickel powder is purchased from Hebei Xinda Alloy Materials (China). To obtain nickel-doped polyimide film, we first mix the polyimide precursor solution and nickel powder (particle diameter of 100 nm) according to a certain proportion and ultrasonically stirred for 0.5 h to obtain the uniform mixture. Then an appropriate volume of the mixture is dropped and spin-coated on the quartz glass at a speed of 2,000 rpm and a time of 90 s. After this procedure, the Ti wires (thickness of 10 μm) are pressed inside the mixed solution. By adjusting the rotation speed, the time, and the number of times, different thicknesses of nickel-doped polyimide films can be achieved. Upon curing on a hot plate at 130 °C and imidization in a furnace at 300 °C, the solvent of the mixture is evaporated and a nickel-doped polyimide film with embedded Ti wires is obtained. After the laser carbonization of the Ni-doped polyimide near the Ti wire, this wire is wrapped by the nickel-catalyzed LIG, thus realizing the connection between LIG and Ti wire. Through these pre-reserved wires, the embedded graphene electronics can be connected to the external devices. It is noted that the thickness of nickel-doped polyimide film for resistance measurement and material characterization is 20 μm. For the passive wireless light sensor, a 200-μm film is prepared. After this procedure, a layer of epoxy is deposited on the surface of the nickel-doped polyimide film.

### Selection of laser sources

To fabricate embedded circuits inside multilayer structures, the laser wavelength and the optical properties of the material must be carefully considered. For the carbon dioxide laser that is commonly used for LIG preparation, the transmittance of most materials with thickness larger than 100 μm will drop below 50%, resulting in inefficient carbonization and poor electrical property. Many materials with wavelengths from 500 to 2,000 nm have transmittances of up to 90% or more, such as sapphire, alumina, magnesium fluoride, soda lime glass, and quartz glass [48]. Therefore, the selection of lasers in this band is more suitable for the fabrication of embedded circuits.

### Fabrication process of rigid circuits

The fabricated multilayer sample (10% nickel content) is first placed on the moving stage. Then, the predesigned pattern is input into a computer to design the laser path. Finally, the processing parameter (power density of 5,570 W/cm^2^ and scanning speed of 1 mm/s) is selected to fabricate the embedded resistor, conductor, inductor, and LC combination circuits.

### Fabrication process of flexible circuits

For the flexible electronics, a sapphire substrate is first selected to deposit a layer of PZT with a thickness of 1 μm (see the detailed procedures in our previous work [49]). Then, nickel-doped polyimide (10% nickel content) and commercial VHB tapes are deposited sequentially on the surface of the PZT. Next, an 808-nm laser source (power density of 3,183 W/cm^2^ and scanning speed of 1 mm/s) is adopted to prepare the embedded graphene circuits. Finally, a 308-nm excimer laser is selected to peel off the rigid sapphire substrate. The exfoliation processing parameter (energy density of 150 mJ/cm^2^, frequency of 10 Hz, and velocity of 0.6 mm/s) is chosen.

### Fabrication process of amplifier circuit

Patterned copper electrodes are first deposited onto a 10% nickel-doped polyimide/quartz glass substrate by magnetron sputtering. Then, various electronic components are assembled to the corresponding position with conductive silver paste. Next, the patterned VHB tape (purchased from 3M Corporation, USA) adheres to a nickel-doped polyimide/quartz glass substrate and the 2 interconnect holes are filled with conductive silver paste. Finally, another nickel-doped polyimide/quartz glass substrate is glued to the VHB tape.

### Material characterization and testing

After carbonization, the LIG is located inside multilayer structures. To characterize and test the properties of LIG, the processed sample is dropped with a peptizer (Shenzhen Zhiwei Technology Co., Ltd, China) to remove epoxy. A scanning electron microscope (SEM, SU8020, Hitachi, Japan) is selected to investigate the morphology of polyimide after carbonization. Raman spectroscopy of carbonization product is performed using a Raman spectrometer (Renishaw inVia Reflex, UK) excited with a 532-nm laser. The height map is characterized by a laser scanning confocal microscope (VK-X200K, Japan). The transmittance and absorption of polyimide films with different nickel content and the absorption of PZT are measured by a ultraviolet spectrophotometer (Lambda 35, USA). A digital external meter (Keysight 34460A, China) is used to measure the resistance of polyimide after carbonization. The crystal lattices of carbonization products with different nickel contents are tested by XRD (XRD-6100, Japan). An x-ray photoelectron spectrometer (AXIS-ULTRA DLD-600W, Kratos, Japan) is used to measure the elemental composition and content of polyimide before and after carbonization. The mass change of polyimide and nickel-doped polyimide with temperature is measured by a thermogravimetric analyzer (TGA8000, PerkinElmer, USA) with a heating rate of 10 °C/min in a nitrogen environment. An LCR meter (Keysight E4980A, China) is selected to measure the resistance and capacitance. A vector network analyzer (Keysight E5063A ENA, China) is used to measure the resonant frequency of the device with the frequency. It is noted that all tests are carried out in a clean room with a constant temperature of 25 °C and relative humidity of 60%.

### COMSOL Multiphysics simulation

The commercial COMSOL Multiphysics simulation software is used to study the temperature distribution of the sample during LaserIW processing with the radiation heat transfer module, solid heat transfer module, and 2 differential equation modules. Among them, the radiation heat transfer module is used for the analysis of absorbing laser radiation energy. The thermal conduction analysis of multilayer structure is investigated by the solid heat transfer module. As for the remaining 2 differential equation modules, they are used to analyze the decomposition of the internal polyimide and epoxy resin layer, respectively. The density, thermal conductivity, and specific heat capacity of quartz glass, polyimide, and epoxy are listed in Table S1 in detail. To simplify the calculation, the overall size of the model is 4,000×2,000×2,020 μm, where the thickness of the quartz glass and epoxy resin are both 1,000 μm and the thickness of polyimide is 20 μm.

### Reactive molecular dynamics simulation

The LAMMPS package integrated with the ReaxFF potential that contains the elements of C, H, O, N, and Ni is used in this study [50]. In order to build the initial system model, the model of polyimide (C_29_H_16_O_6_N_2_) monomers is first built and geometrically optimized; and then 6 polyimide monomers and 6 nickel atoms are assigned into a cell. After that, the NVT ensemble and periodic boundary conditions are applied to the cells with a timestep of 0.25 femtosecond (fs). Then the system is kept at 3,000 K controlled by the Nose-Hoover thermostat method for 400 ps. To study the effect of nickel on the carbonization of polyimide, the nickel atoms in the above model are removed to calculate the carbonization result of pure polyimide again while other parameters remain unchanged. The open-source OVITO package is used for the post-processing of molecular dynamics simulation results.

## Data Availability

The data are available from the authors upon a reasonable request.

## Supplementary Materials

Supplementary 1 Movies S1 and S2 Figs. S1 to S24 Table S1 References [51–56]
