# Supplementary material for: Laser-Guided, Self-Confined Graphitization for High-Conductivity Embedded Electronics
Source: Research (Wash D C). 2024 Feb 12;7:0305. doi: 10.34133/research.0305 (PMC11020139; doi:10.34133/research.0305)
Supplement: Supplementary 1 — Movies S1 and S2 Figs. S1 to S24 Table S1 References [51–56] [file research.0305.f1.docx]

Supporting Information Text

### Heat transfer and chemical decomposition of the theoretical model

A photothermal model was established to investigate the relationship between the temperature distribution of laser interface-confined ablation and the absorbed laser energy. Owing to the transparency of quartz glass to 808 nm laser, the transmitted laser is absorbed by the interfacial polyimide. According to the Beer-Lambert law [51], the heat source density at the position (x, y, z) in the interfacial polyimide film is:

$Q_{1}=\alpha(1-\mathrm{R})\frac{2P}{\pi r_{spot}^{2}}\exp(-\frac{2(x^{2}+y^{2})}{r_{spot}^{2}})\exp(-\alpha z)$ (1)

where α and R are the absorption coefficient and reflectivity of polyimide, respectively [39]. P and r_spot_ represent the laser power and spot radius, respectively. During laser irradiation, the temperature of the interfacial polyimide film rises. Once the critical temperature is reached, chemical decomposition reactions will occur [52, 53]. In this study, a chemical decomposition kinetics model was built to describe this process [54], which is described as follows:

$\frac{\partial n_{p}}{\partial t}=(1-n_{p})\mathrm{c}_{p}\exp(-\frac{E_{p}}{k_{B}T_{p}})$ (2)

$\frac{\partial n_{e}}{\partial t}=(1-n_{e})\mathrm{c}_{e}\exp(-\frac{E_{e}}{k_{B}T_{e}})$ (3)

Equations for the heat absorbed by the chemical decomposition of polyimide and epoxy resin are expressed as:

$Q_{2}\text{=}\Delta\mathrm{H}_{p}N_{p}(1-n_{p})\mathrm{c}_{p}\exp(-\frac{E_{p}}{k_{B}T_{p}})$ (4)

$Q_{e}=\Delta\mathrm{H}_{e}N_{e}(1-n_{e})\mathrm{c}_{e}\exp(-\frac{E_{e}}{k_{B}T_{e}})$ (5)

Based on the above heat source density, the transient variation of the temperature distribution T (x, y, z, t) of the quartz glass, interfacial polyimide, and epoxy can be obtained from the classical heat transfer equation:

$\rho_{q}(C_{p})_{q}\frac{\partial T_{q}}{\partial t}-\frac{\partial}{\partial x}(\mathrm{k}_{q}\frac{\partial T_{q}}{\partial x})-\frac{\partial}{\partial y}(\mathrm{k}_{q}\frac{\partial T_{q}}{\partial y})-\frac{\partial}{\partial z}(\mathrm{k}_{q}\frac{\partial T_{q}}{\partial z})=0$ (6)

$\rho_{p}(C_{p})_{p}\frac{\partial T_{p}}{\partial t}-\frac{\partial}{\partial x}(\mathrm{k}_{p}\frac{\partial T_{p}}{\partial x})-\frac{\partial}{\partial y}(\mathrm{k}_{p}\frac{\partial T_{p}}{\partial y})-\frac{\partial}{\partial z}(\mathrm{k}_{p}\frac{\partial T_{p}}{\partial z})=Q_{1}\text{-}Q_{2}$ (7)

$\rho_{e}(C_{p})_{e}\frac{\partial T_{e}}{\partial t}-\frac{\partial}{\partial x}(\mathrm{k}_{e}\frac{\partial T_{e}}{\partial x})-\frac{\partial}{\partial y}(\mathrm{k}_{e}\frac{\partial T_{e}}{\partial y})-\frac{\partial}{\partial z}(\mathrm{k}_{e}\frac{\partial T_{e}}{\partial z})=\text{-}Q_{e}$ (8)

where the subscripts q, p, and e represent quartz glass, polyimide, and epoxy resin, respectively. The *ρ*, C, and k are the density, temperature-dependent specific heat, and thermal conductivity, respectively. When the temperature of polyimide carbonization is reached, the properties of the polyimide change greatly. For simplicity, the variation of the material parameters of polyimide with temperature is represented as a step function in this model. For the extinction coefficient of polyimide, a value of 0.003 is used for temperatures below the critical temperature, and a value of 1.9 is used for temperatures above this critical temperature [55,56]. The temperature-dependent specific heat and thermal conductivity of polyimide are expressed as follows:

$\text{（}C_{p}\text{）}_{p}=1000[0.96+1.39(\frac{T-300}{400})-0.43(\frac{T-300}{400})^{^{2}}]$ (9)

 (10)

The specific heat and thermal conductivity of quartz glass are fixed as 730 J/(kg·K) and 1.4 W/(m·K), respectively. For epoxy resin, its specific heat and thermal conductivity can be expressed as:

 (11)

 (12)

For any point, T_q_=T_p_=T_e_=300 K is at the initial time. Meantime, when *x*, *y*, or *z* is infinite, T_q_=T_p_=T_e_=300 K for any *t*. At the interface of quartz glass and polyimide, there are:

$k_{q}\frac{\partial T_{q}}{\partial x}=k_{p}\frac{\partial T_{p}}{\partial x}$ (13)

$k_{q}\frac{\partial T_{q}}{\partial y}=k_{p}\frac{\partial T_{p}}{\partial y}$ (14)

$k_{q}\frac{\partial T_{q}}{\partial z}=k_{p}\frac{\partial T_{p}}{\partial z}$ (15)

Ignoring the thermal resistance effect, the temperature of the point at the interface is equal. Besides, n_p_=n_e_=0 exists for any position at the initial time.

Table S1 Comparison of LaserIW and existing surface processing techniques

| Various techniques | | Electrical conductivity (S/m) | Structural integrity | Chemical agent | Self-encapsulation | Non-destructive renovation/repair | |
| --- | --- | --- | --- | --- | --- | --- | --- |
| Chemical reduction [28] | 0.25 | | With exfoliation | NaNO_3_, KMnO_4_, HCl, H_2_O_2_, VC | No | | No |
| Plasma reduction [29] | 250 | | With exfoliation and oxidation | H_2_SO_4_, KMnO_4_, HCl, H_2_O_2_ | No | | No |
| Thermal reduction [30] | 1623 | | With oxidation | Cyclohexanone, ethylene glycol, ethyl cellulose, hexa-methyldisilazane, | No | | No |
| Laser reduction [31] | 3125 | | With exfoliation and oxidation | Zn(CH_3_COO)_2_ ∙ 2H_2_O, H_2_SO_4_, KMnO_4_, HCl, H_2_O_2_ | No | | No |
| Surface photochemical graphitization [32] | | 1282 | With exfoliation and oxidation | Polyimide  precursor | No | | No |
| Surface photothermal graphitization [33] | 2500 | | With exfoliation and oxidation | Polyimide  precursor | No | | No |
| LaserIW (this work) | 20000 | | No exfoliation or oxidation | Polyimide  precursor | Yes | | Yes |


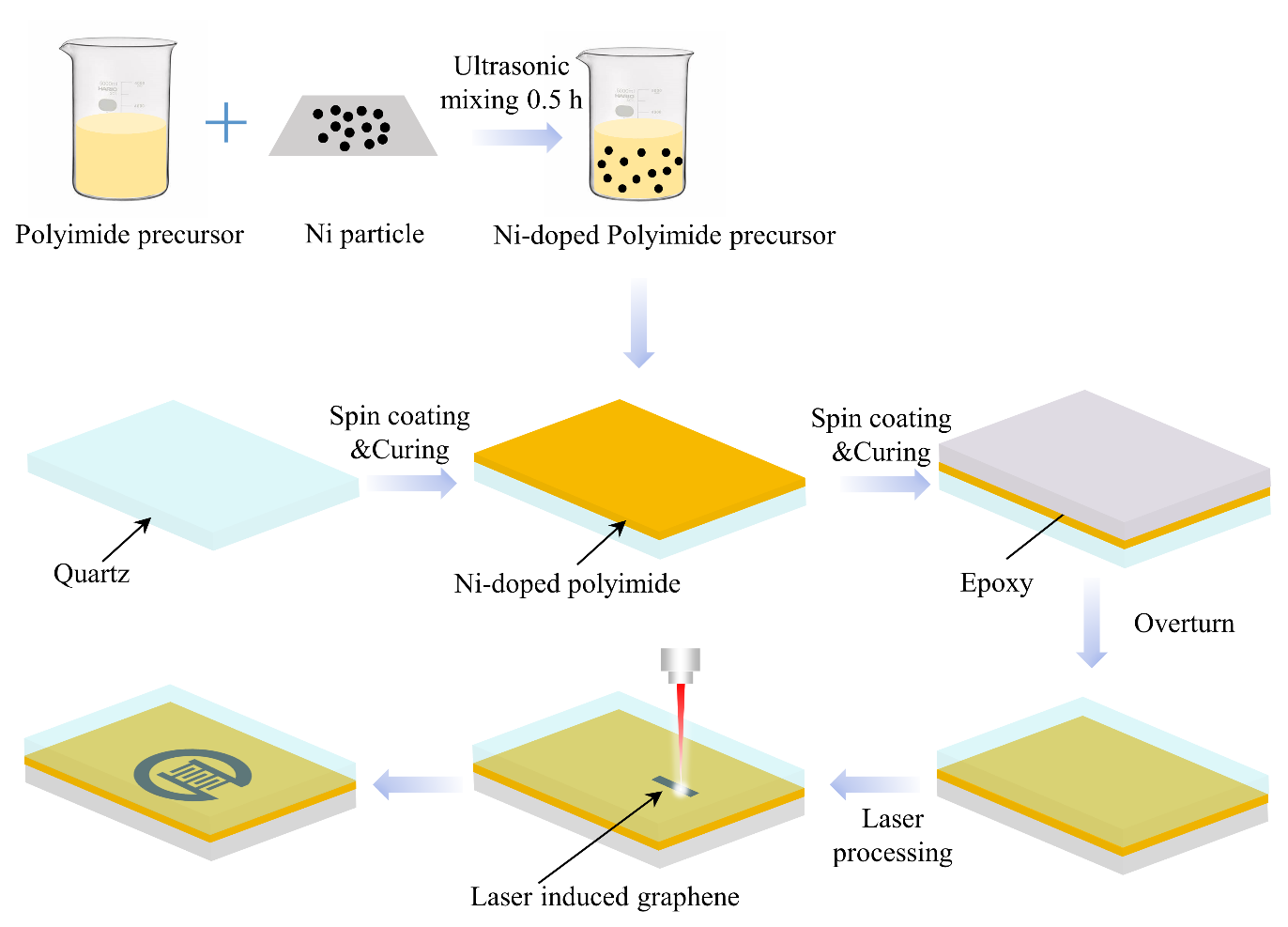


**Figure S1. Preparation process of graphene circuit inside multilayer structures**


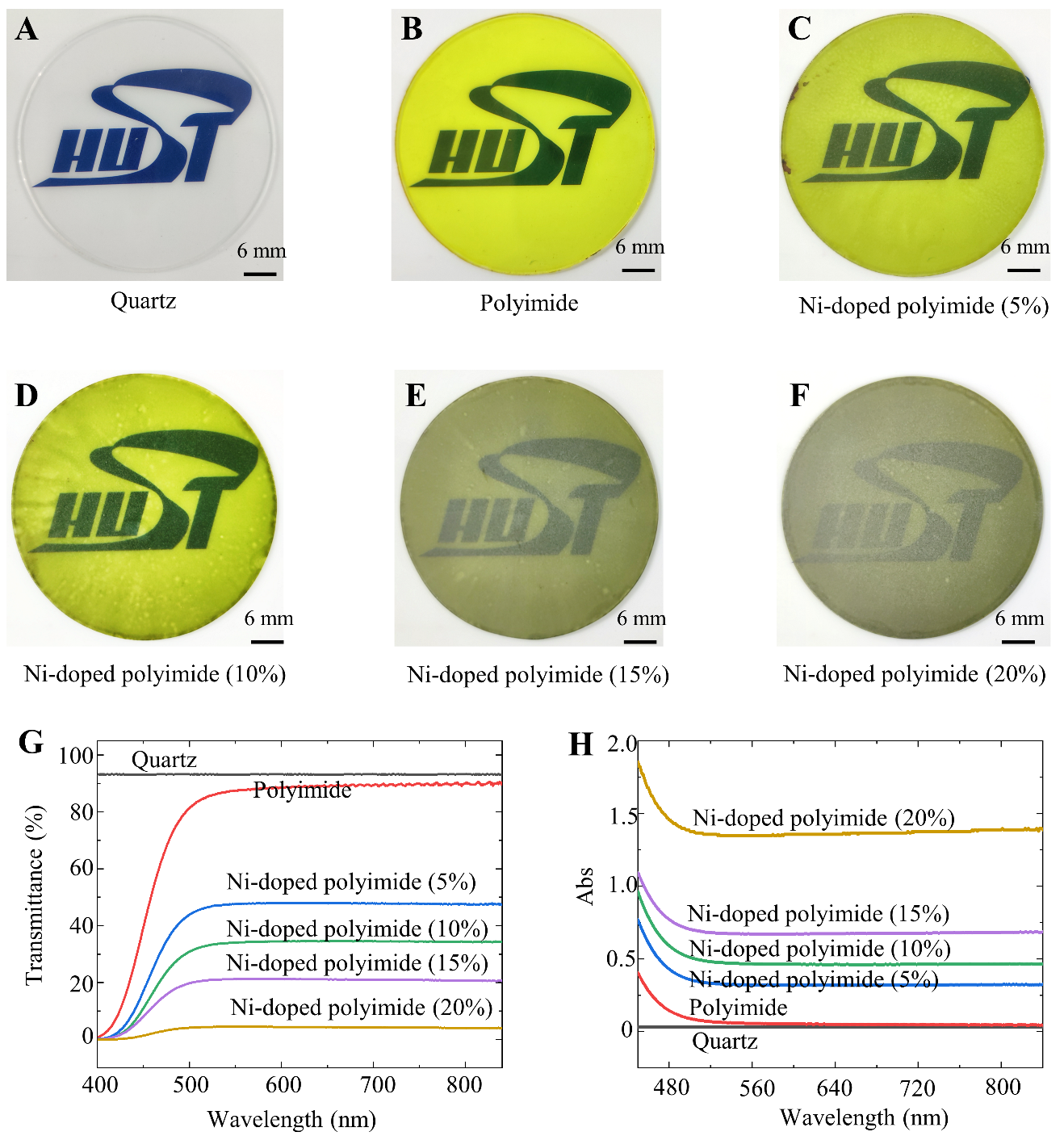


**Figure S2. Optical properties of quartz glass and polyimide with different nickel contents.** **A,** Photograph of quartz glass. **B,** Photograph of pure polyimide. **C,** Photograph of polyimide with 5% nickel content. **D,** Photograph of polyimide with 10% nickel content. **E,** Photograph of polyimide with 15% nickel content. **F,** Photograph of polyimide with 20% nickel content. **G,** Transmittance of quartz glass and polyimide with different nickel contents. **H,** Absorbance of quartz glass and polyimide with different nickel contents.


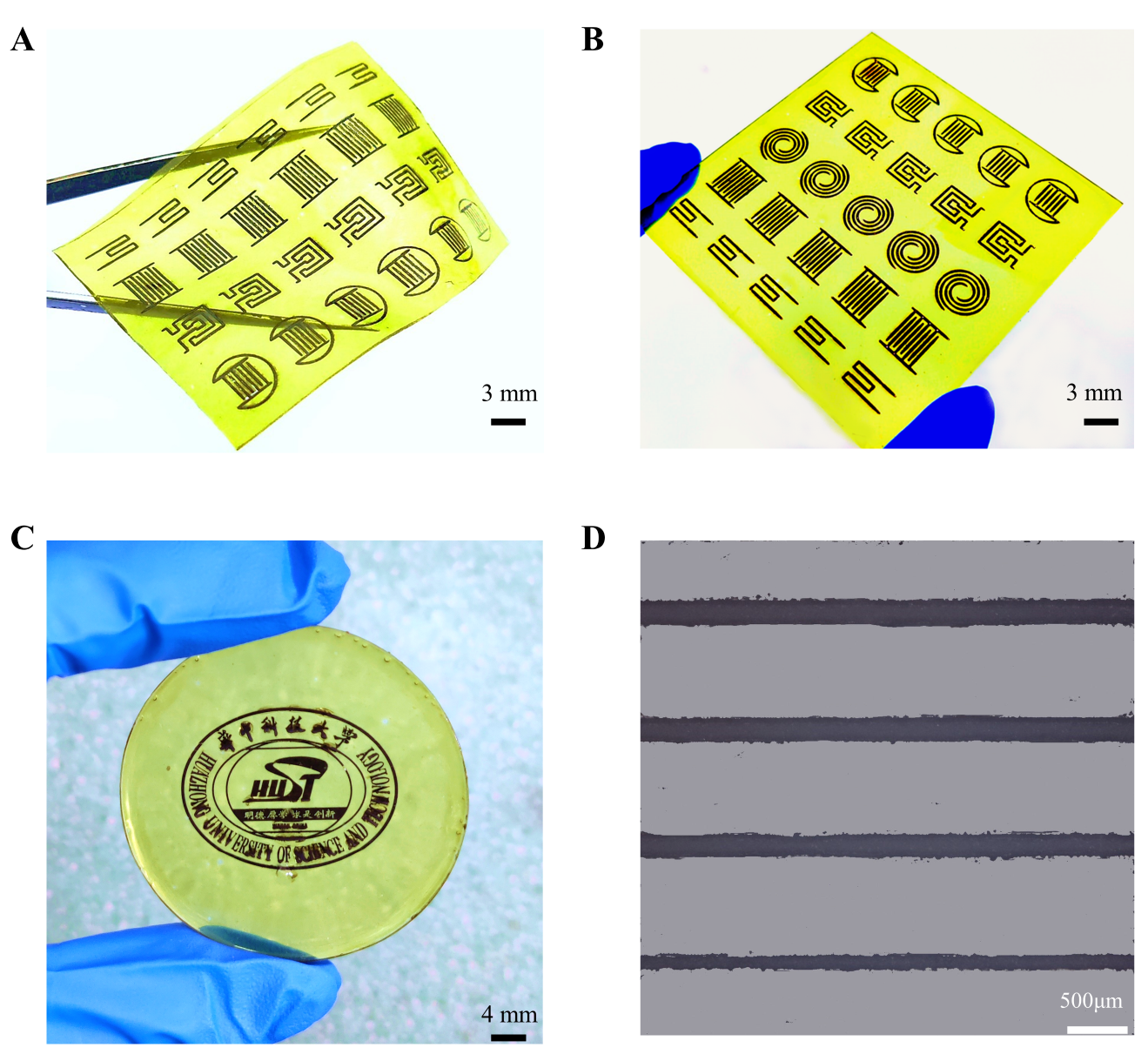


**Figure S3. Various samples and line widths.** Photographs of (**A**) flexible and (**B**) rigid circuit array. **C,** Logo of Huazhong University of Science and Technology. **D,** Various line widths of LIG.


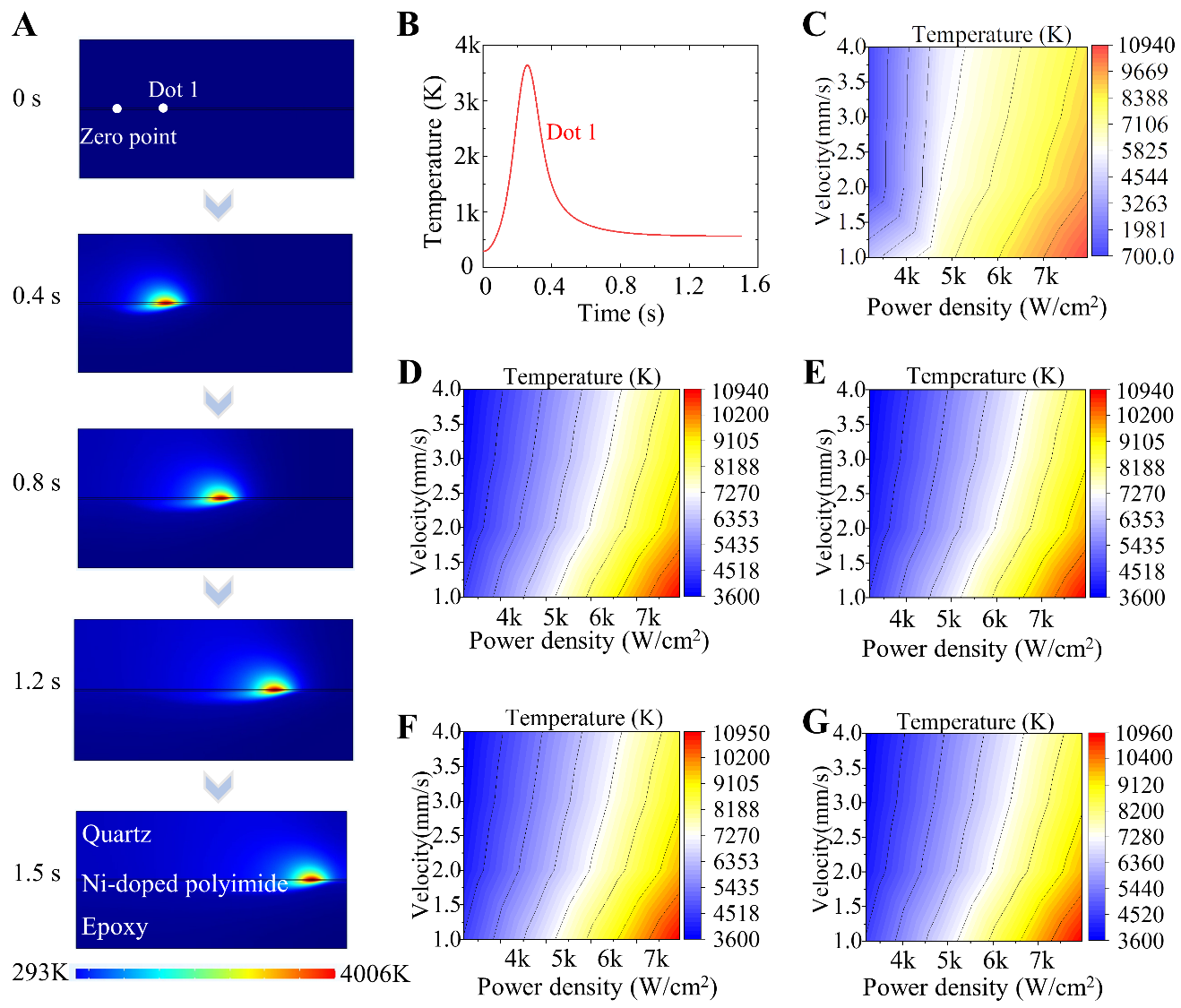


**Figure S4. Temperature analysis. A,** Temperature field distribution at different times. **B,** Variation of temperature at the position of dot 1 over time. **C,** Relationship between the maximum temperature of the pure polyimide and the laser power density and scanning speed. **D,** Relationship between the maximum temperature of the polyimide with 5% nickel content and the laser power density and scanning speed. **E,** Relationship between the maximum temperature of the polyimide with 10% nickel content and the laser power density and scanning speed. **F,** Relationship between the maximum temperature of the polyimide with 15% nickel content and the laser power density and scanning speed. **G,** Relationship between the maximum temperature of the polyimide with 20% nickel content and the laser power density and scanning speed.


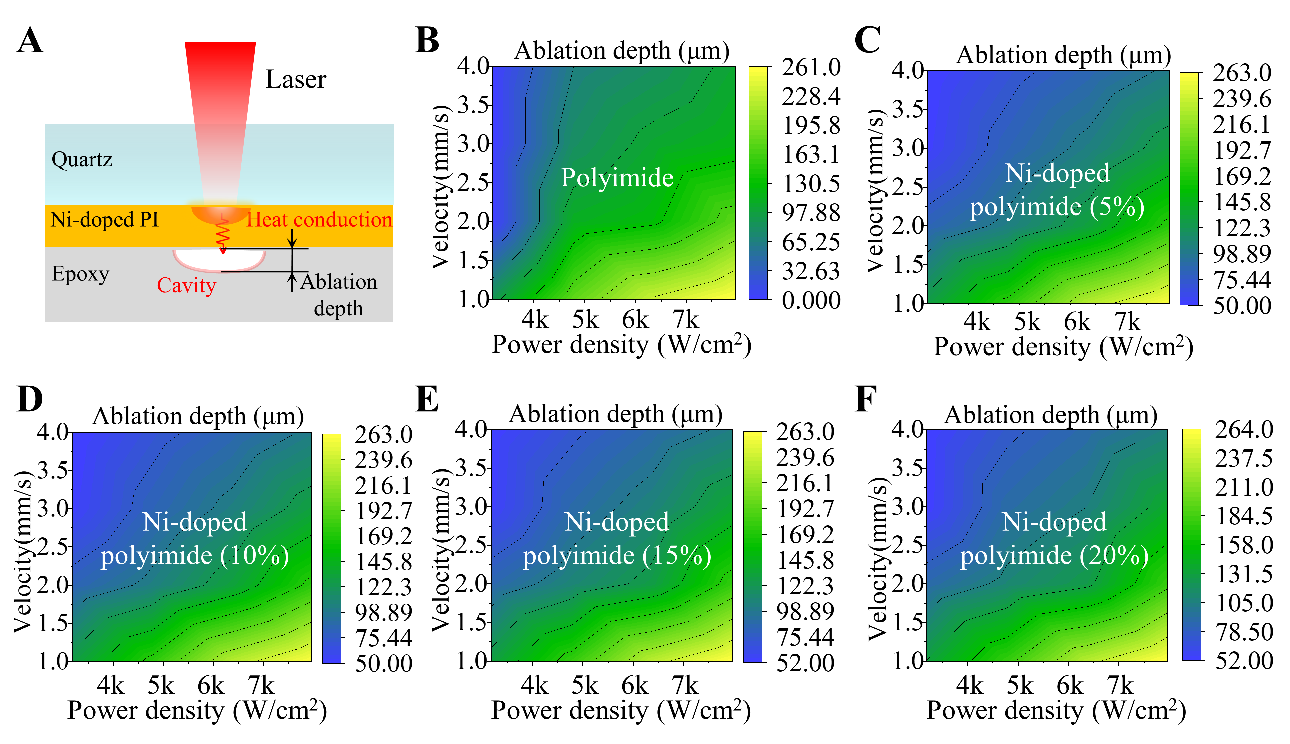


**Figure S5. Ablation depth of epoxy resin. A,** Schematic of epoxy ablation depth. **B,** Relationship between epoxy resin ablation depth and laser power density and scanning speed for pure polyimide. **C,** Relationship between epoxy resin ablation depth and laser power density and scanning speed for polyimide with 5% nickel content. **D,** Relationship between epoxy resin ablation depth and laser power density and scanning speed for polyimide with 10% nickel content. **E,** Relationship between epoxy resin ablation depth and laser power density and scanning speed for polyimide with 15% nickel content. **F,** Relationship between epoxy resin ablation depth and laser power density and scanning speed for polyimide with 20% nickel content.


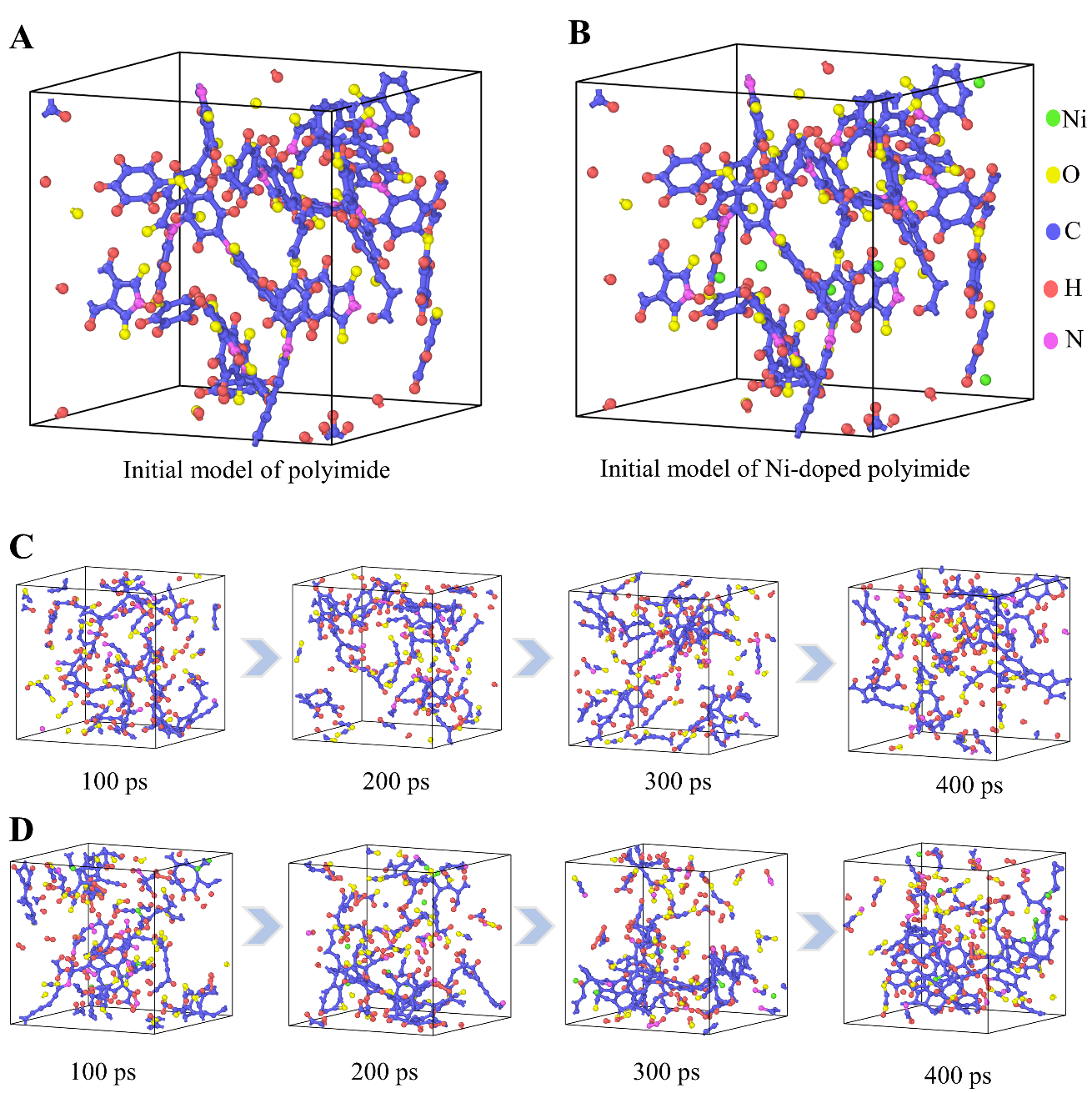


**Figure S6. Molecular dynamics simulation analysis. A,** Initial model of pure polyimide. **B,** Initial model of nickel-doped polyimide. **C,** Decomposition process of pure polyimide at 3000 K. **D,** Decomposition process of nickel-doped polyimide at 3000 K.


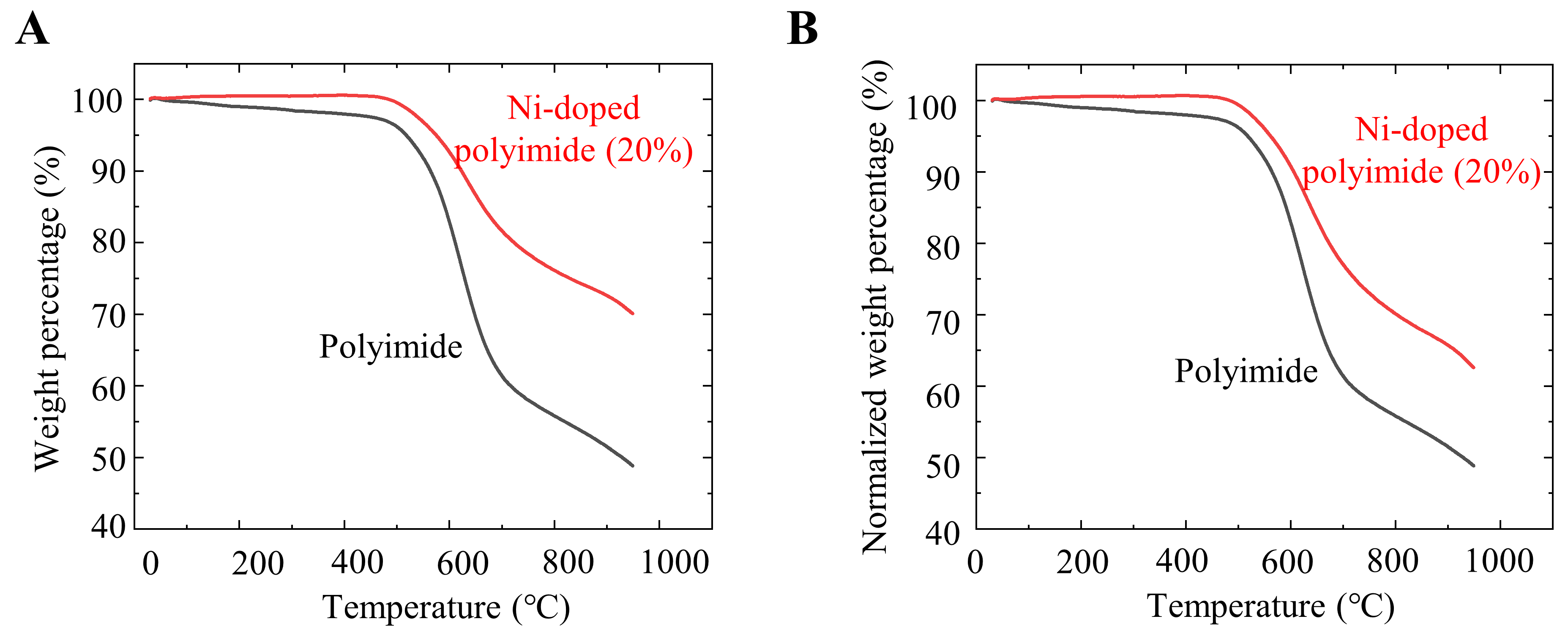


**Figure S7. Thermogravimetric analysis of the pure polyimide and nickel-doped polyimide. A,** Relationship between weight percentage and temperature of pure polyimide and polyimide with 20% nickel content. **B,** Normalized weight percentage of pure polyimide and polyimide with 20% nickel versus temperature


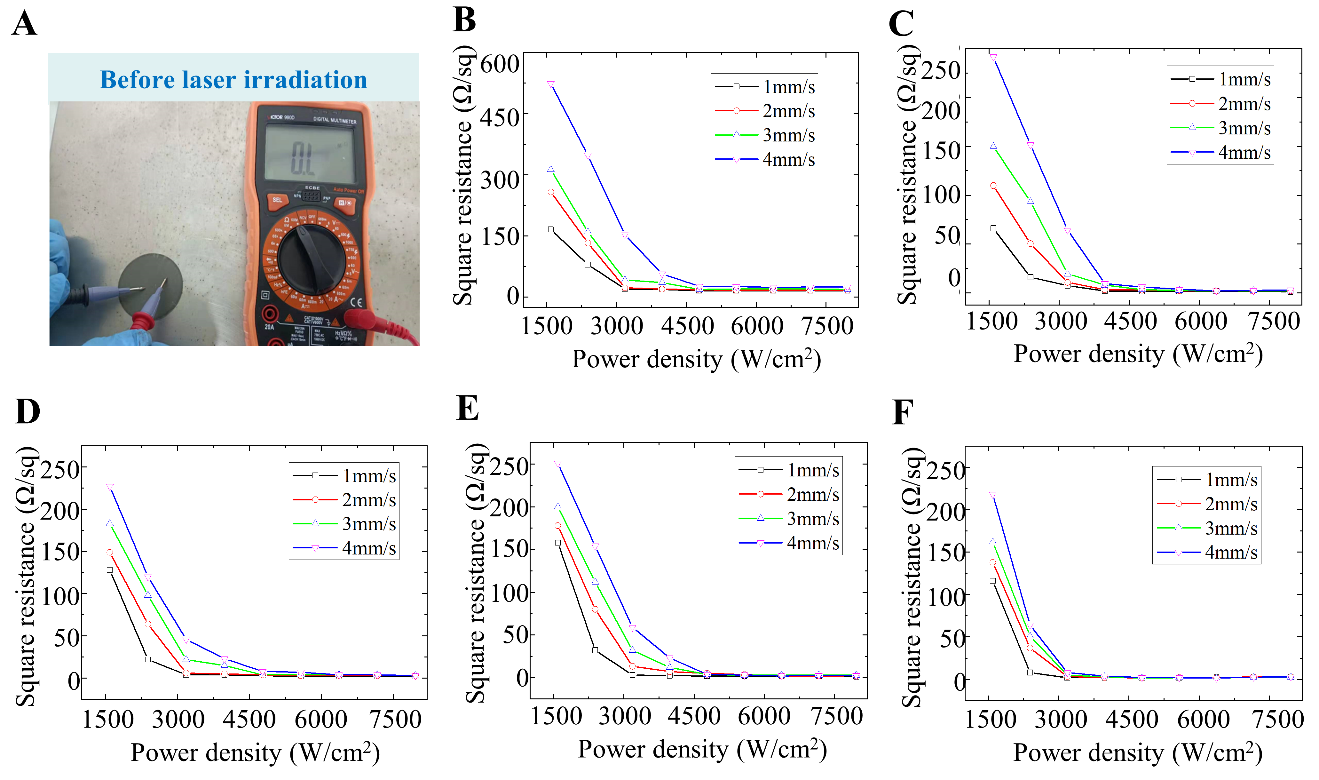


**Figure S8. Resistance test of LIG. A,** Resistance test of the polyimide with 20% nickel content. **B,** Relationship between LIG carbonized by pure polyimide and laser power density and scanning speed. **C,** Relationship between LIG carbonized by polyimide with 5% nickel content and laser power density and scanning speed. **D,** Relationship between LIG carbonized by polyimide with 10 % nickel content and laser power density and scanning speed. **D,** Relationship between LIG carbonized by polyimide with 15% nickel content and laser power density and scanning speed. **F,** Relationship between LIG carbonized by polyimide with 20% nickel content and laser power density and scanning speed


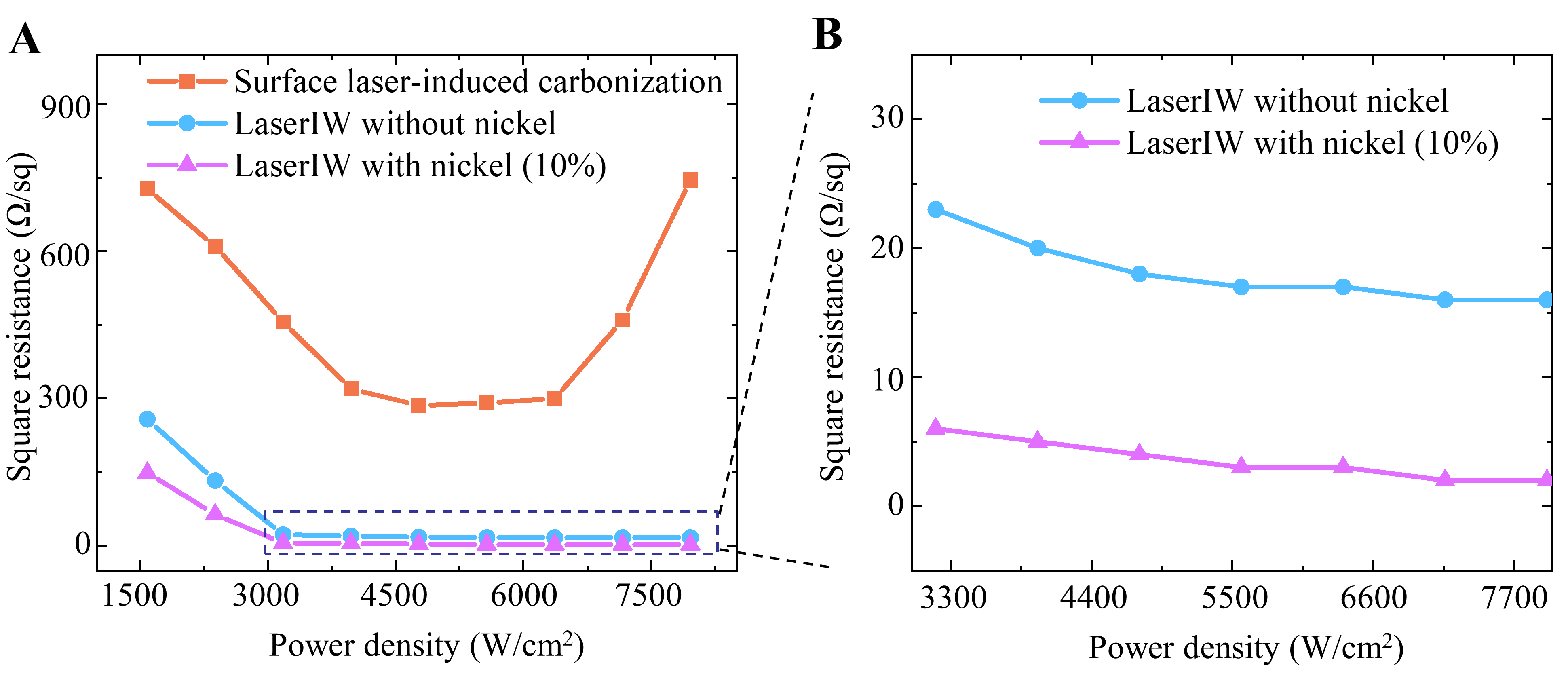


**Figure S9. Resistance comparison of** **samples fabricated by LaserIW and** **surface laser-induced carbonization techniques at 3183 W/cm^2^ and 2 mm/s. A,** Correlation of square resistance with power density for samples fabricated by LaserIW and surface laser-induced carbonization techniques. **B,** The enlarged view of the marked area in (A) shows the significant effect of nickel on the square resistance.


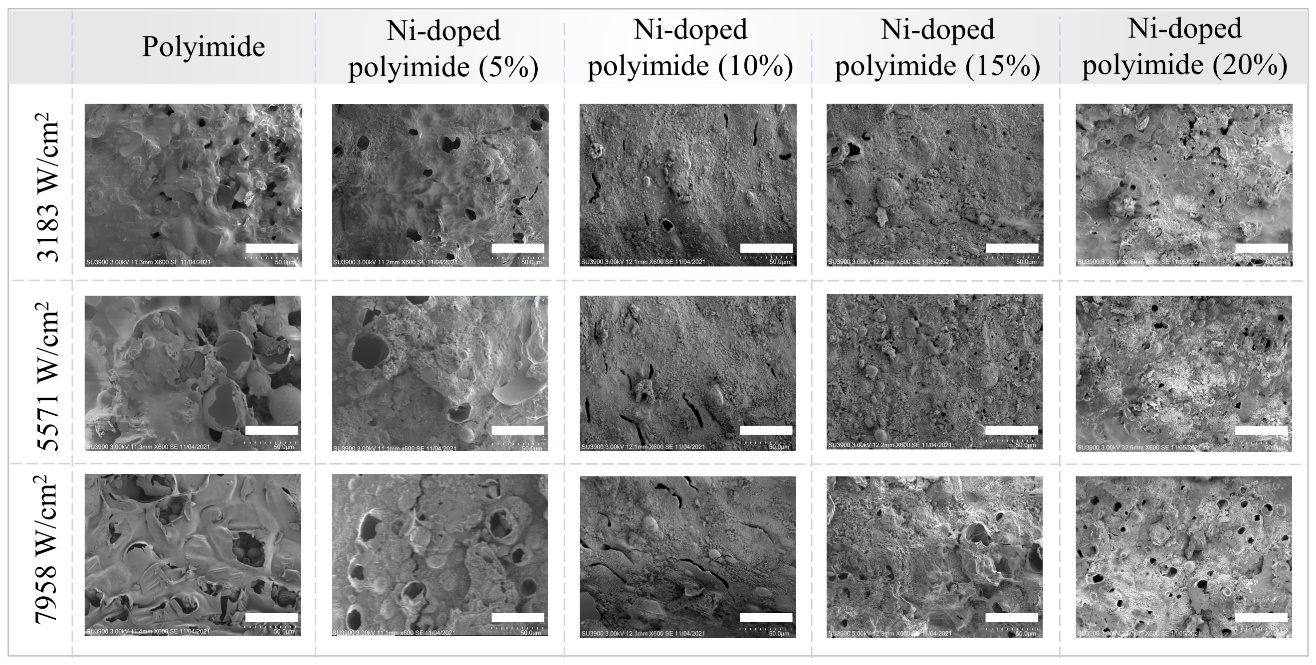


**Figure S10. Effects of laser power and nickel content on the surface morphology at 2 mm/s**


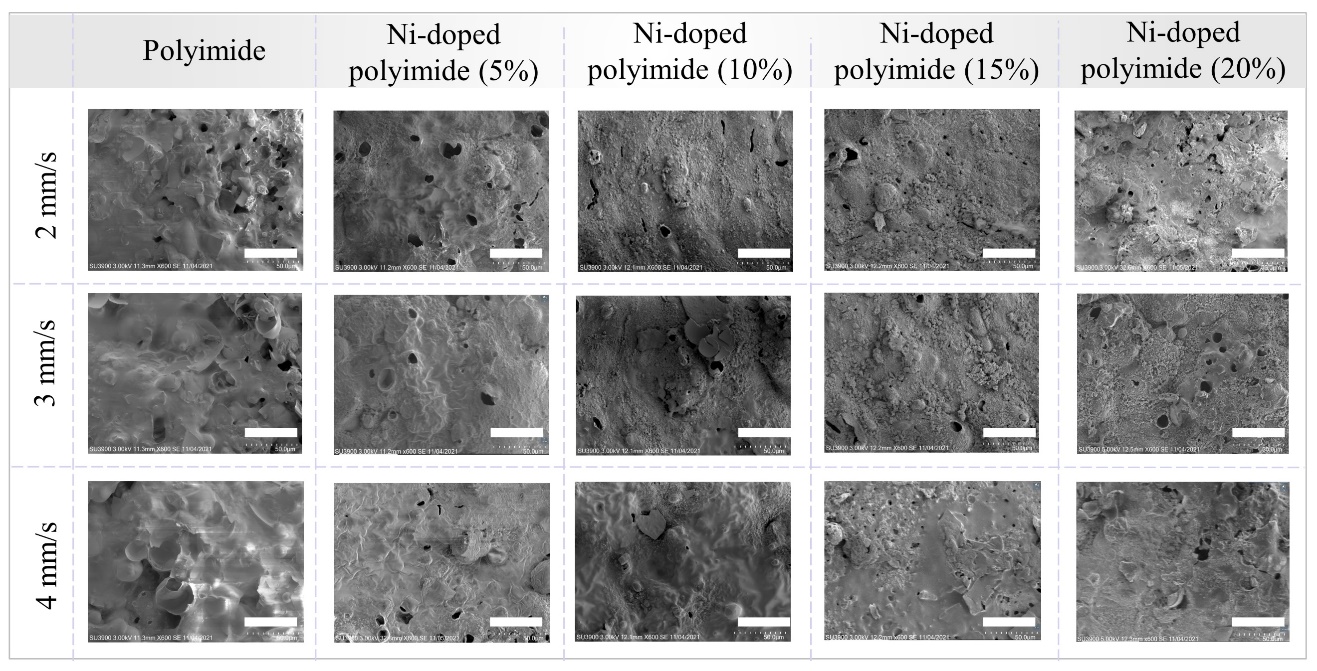


**Figure S11. Effects of scanning speed and nickel content on the surface morphology at 3183 W/cm^2^**


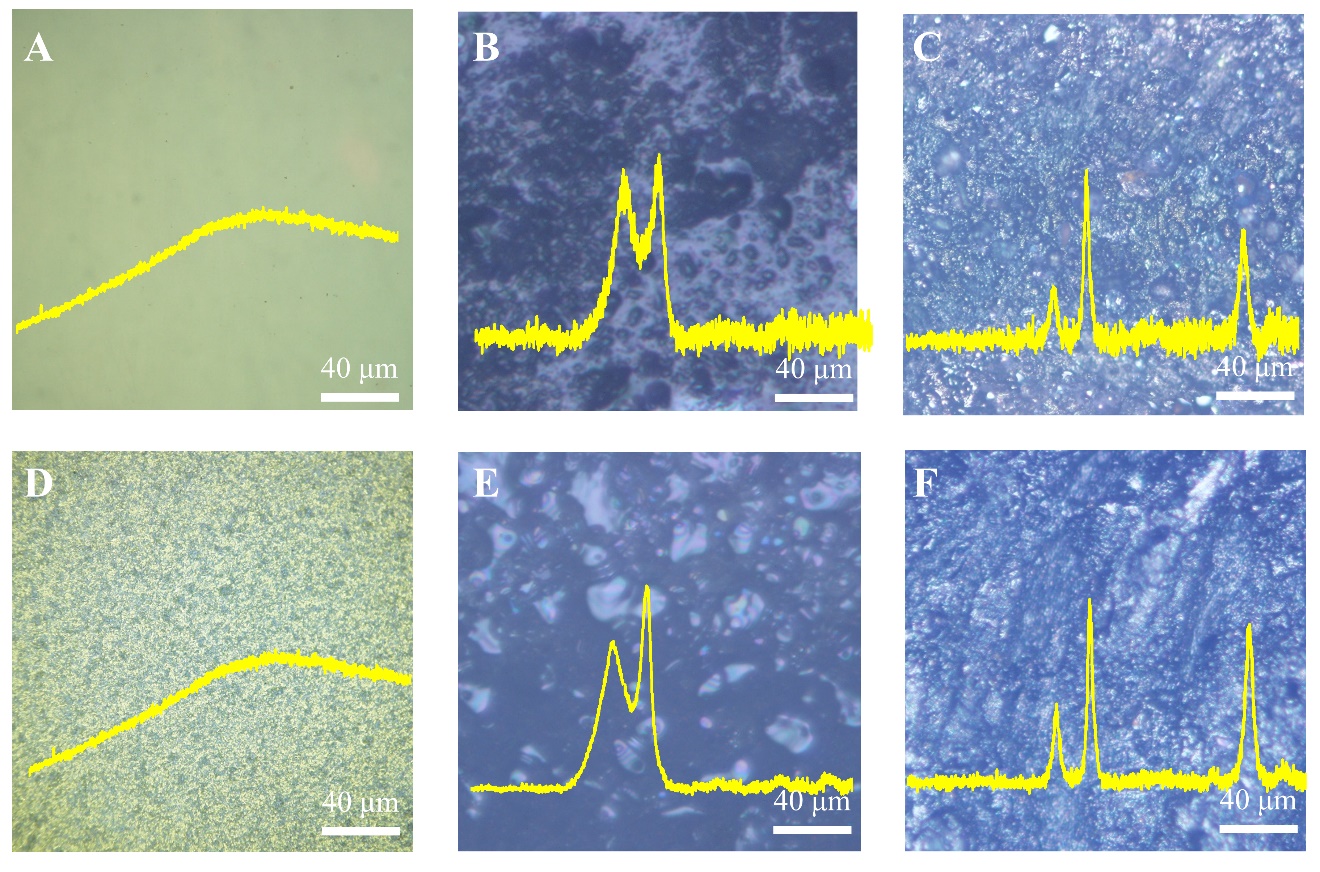


**Figure S12. Effects of laser power and nickel content on** **Raman test results. A,** Surface morphology and Raman test result of LIG at 3183 W/cm^2^ and 2 mm/s. **B,** Surface morphology and Raman test result of LIG at 5570 W/cm^2^ and 2 mm/s. **C,** Surface morphology and Raman test result of LIG at 7958 W/cm^2^ and 2 mm/s. **D,** Surface morphology and Raman test result of Ni-catalyzed LIG at 3183 W/cm^2^ and 2 mm/s. **E,** Surface morphology and Raman test result of Ni-catalyzed LIG at 5570 W/cm^2^ and 2 mm/s. **F,** Surface morphology and Raman test result of Ni-catalyzed LIG at 7958 W/cm^2^ and 2 mm/s.


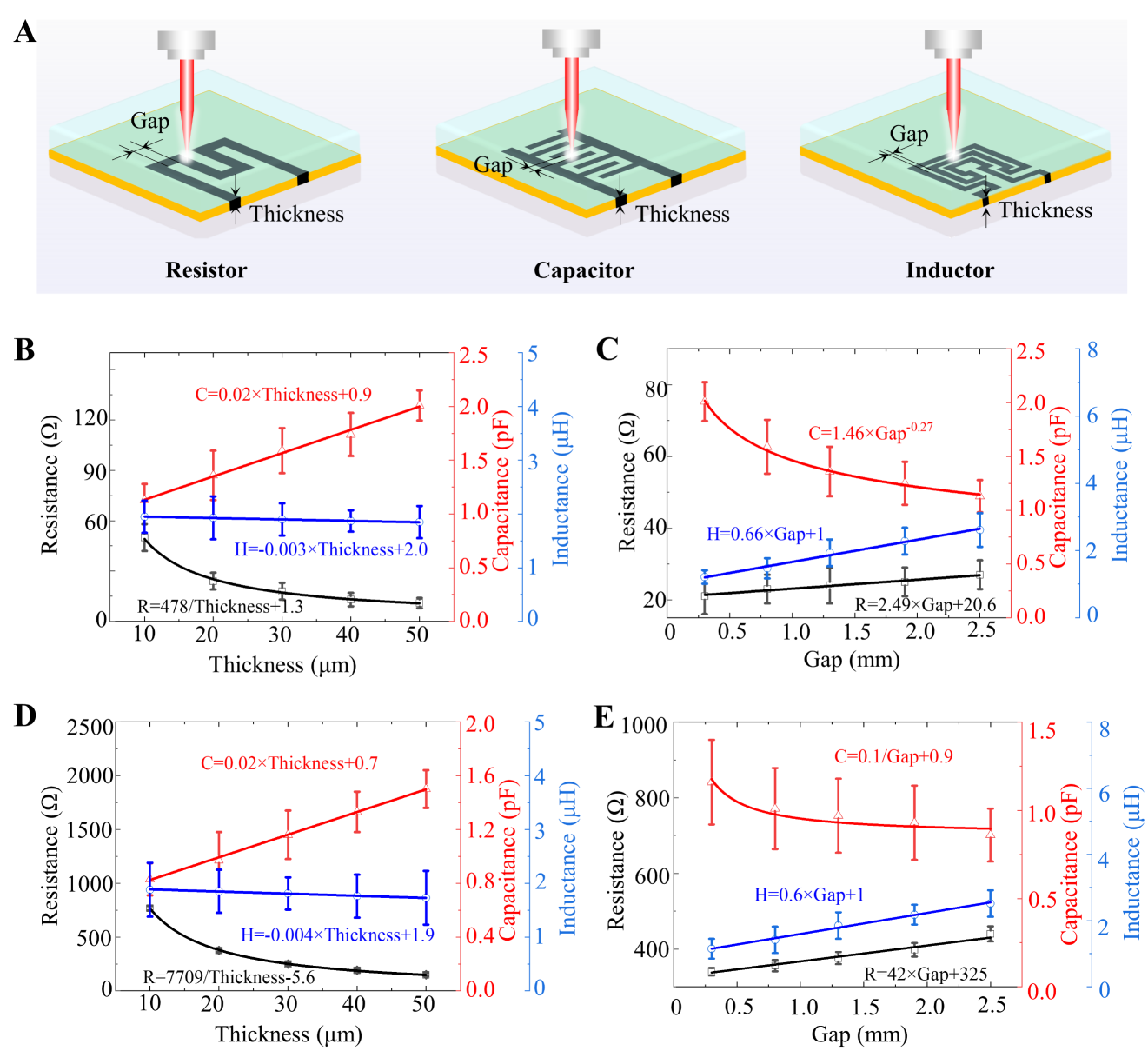


**Figure S13. Fabrication of different electronic devices by the LaserIW technique.** Correlation of component performance with (**A**) thickness and (**B**) gap for nickel-doped polyimide. Relationship between component performance and (**C**)thickness and (**D**) gap for pure polyimide.


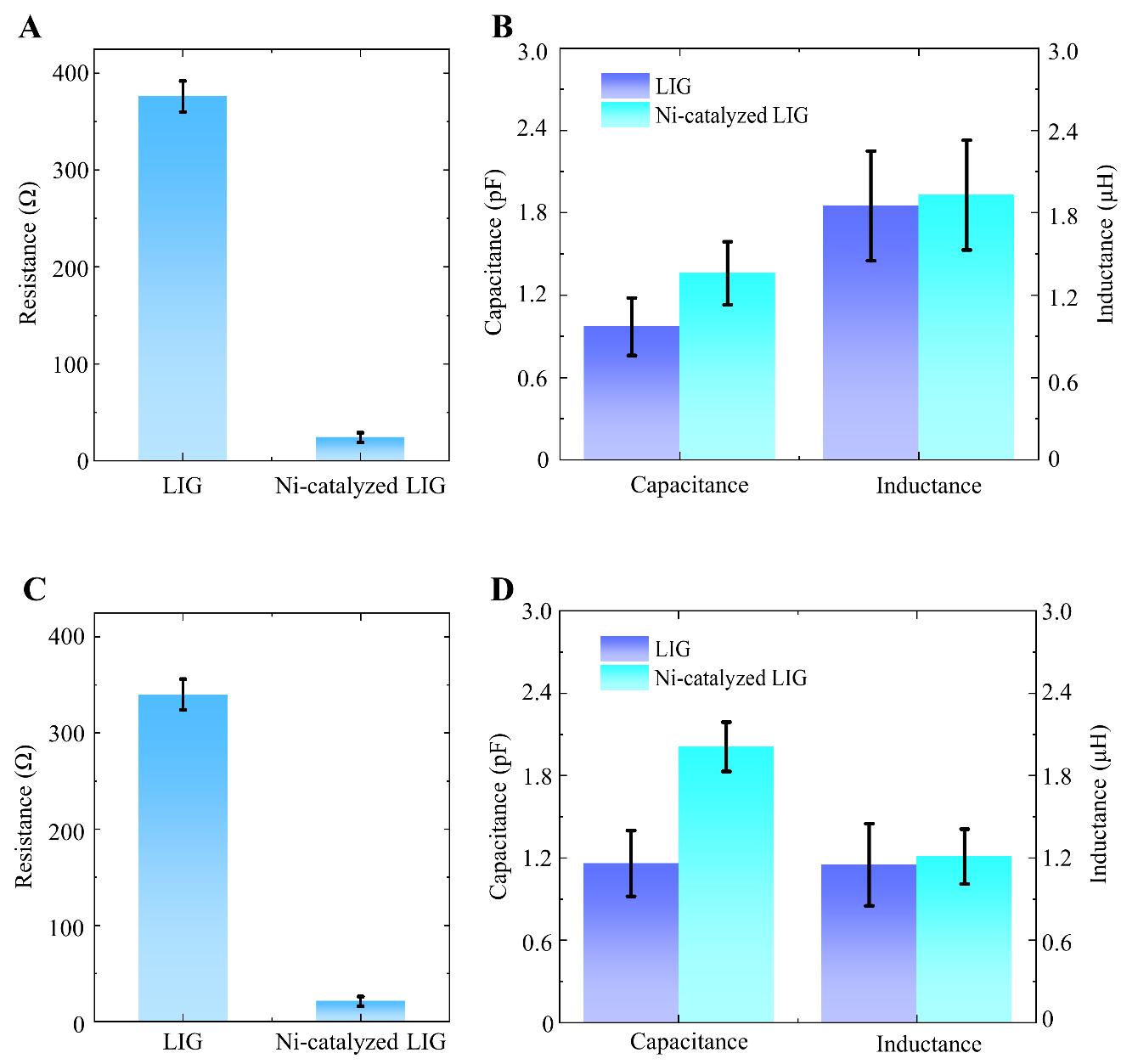


**Figure S14. Performance comparison of LIG and nickel-catalyzed LIG by the LaserIW technique. A,** Resistance comparison under the same gap of 1.3 mm. **B,** Comparison of capacitance and inductance under the same parallel line gap of 1.3 mm. **C,** Resistance comparison under the same thickness of 20 μm. **D,** Comparison of capacitance and inductance under the same thickness of 20 μm.


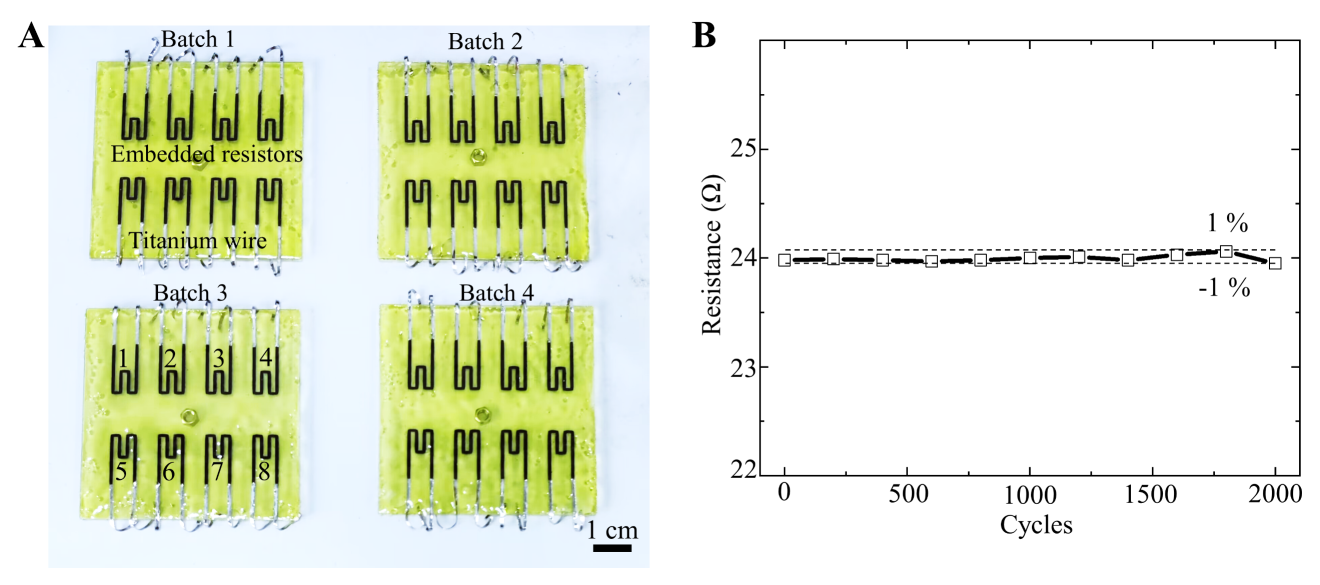


**Figure S15. Reproducible and reliable tests of the embedded graphene electronics. A,** Photograph of the four sample batches. **B,** Resistance variation under different cycles.


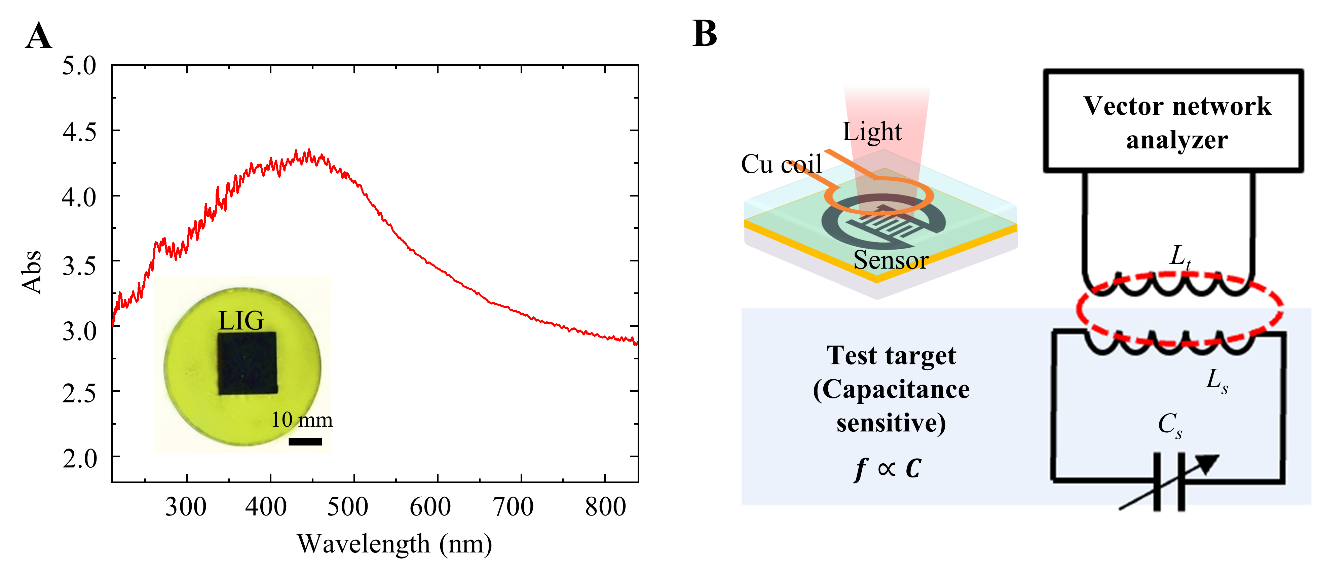


**Figure S16. Performance test of LIG and its application for wireless passive light sensor. A,** Correlation of resistance variation rate with temperature for LIG-based resistors. **B,** schematic diagram for LIG-based passive light sensor


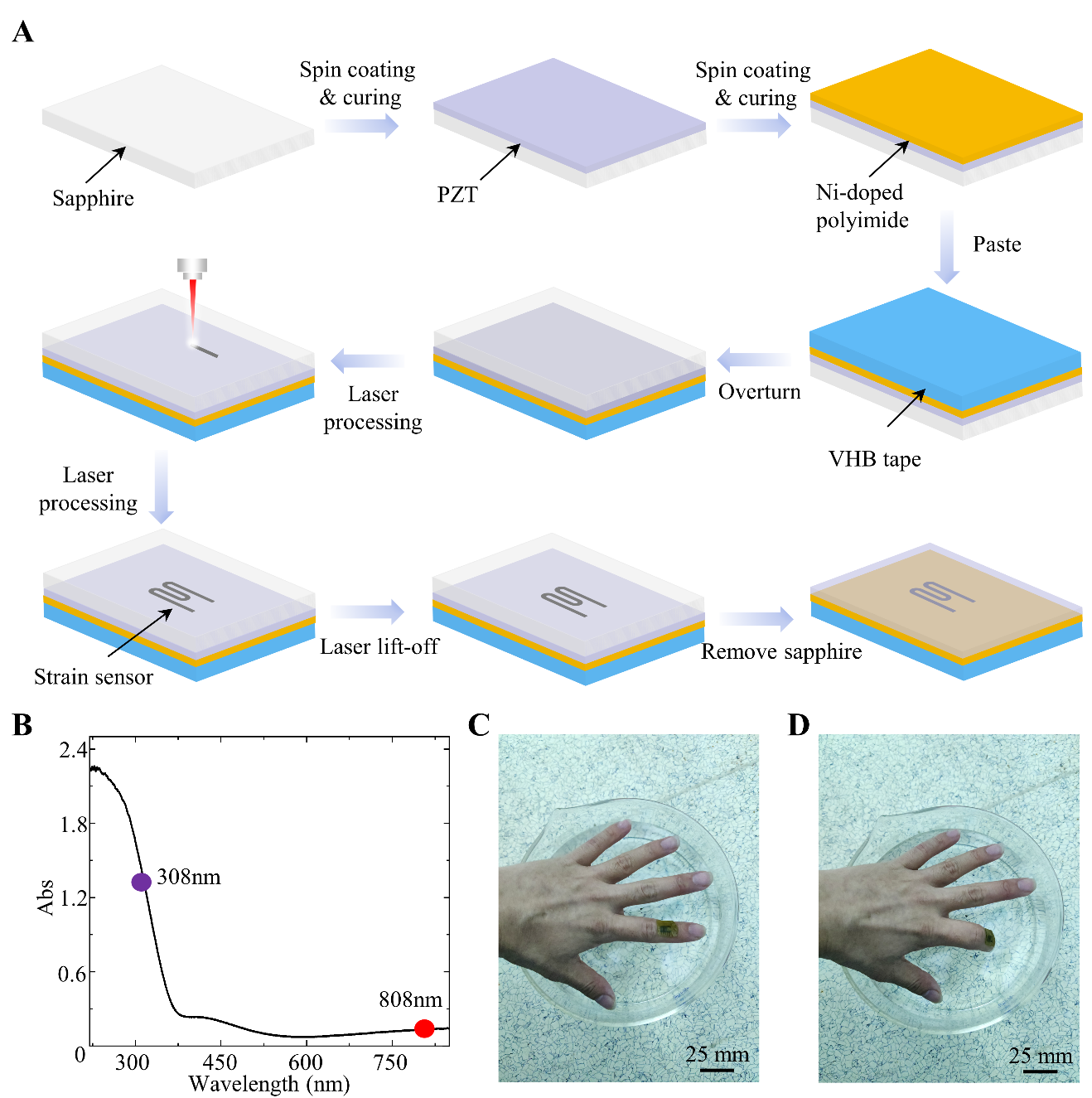


**Figure S17. Design of flexible devices by the LaserIW technique. A,** Fabrication process of flexible electronics devices. **B,** Transmittance of PZT at different wavelengths_._ **C,** Photograph of initial finger state. **D,** Photograph of a bent finger.


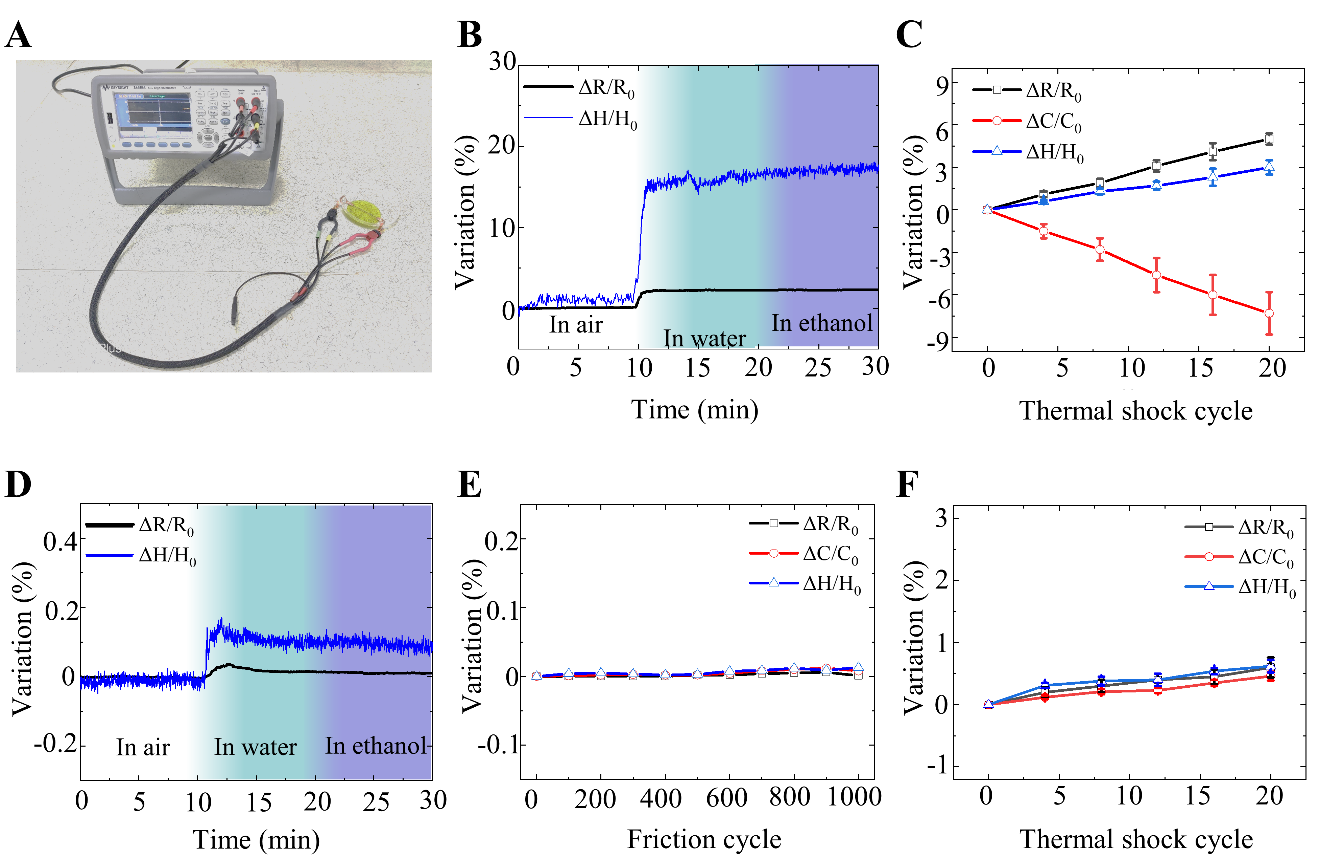


**Figure S18. Self-encapsulation performance test of components fabricated by LaserIW and surface laser-induced carbonization techniques. A,** Test equipment**. B,** Liquid-proof performance test of a device by the surface laser-induced carbonization technique. **C,** Wear-proof performance test of a device by the surface laser-induced carbonization technique. **D,** Thermal shock test of a device by the LaserIW technique. **E,** Liquid-proof performance test of the device by the LaserIW technique. **F,** Thermal shock performance test of the device by the LaserIW technique.


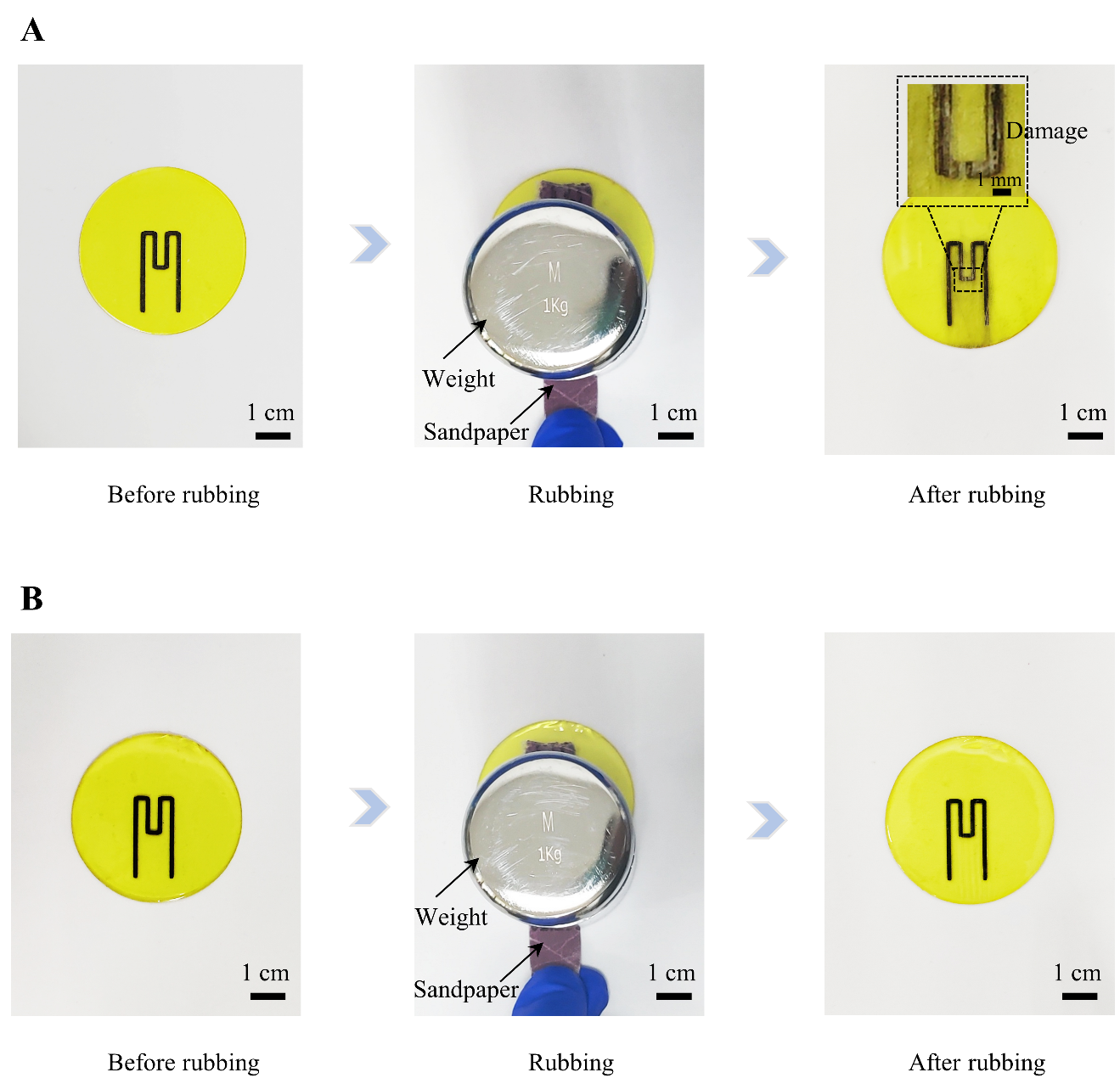


**Figure S19. Anti-friction test of components by LaserIW and surface laser-induced carbonization techniques. A,** Wear-proof test of a sample by the LaserIW technique. **B,** Wear-proof test of a sample by surface laser-induced carbonization.


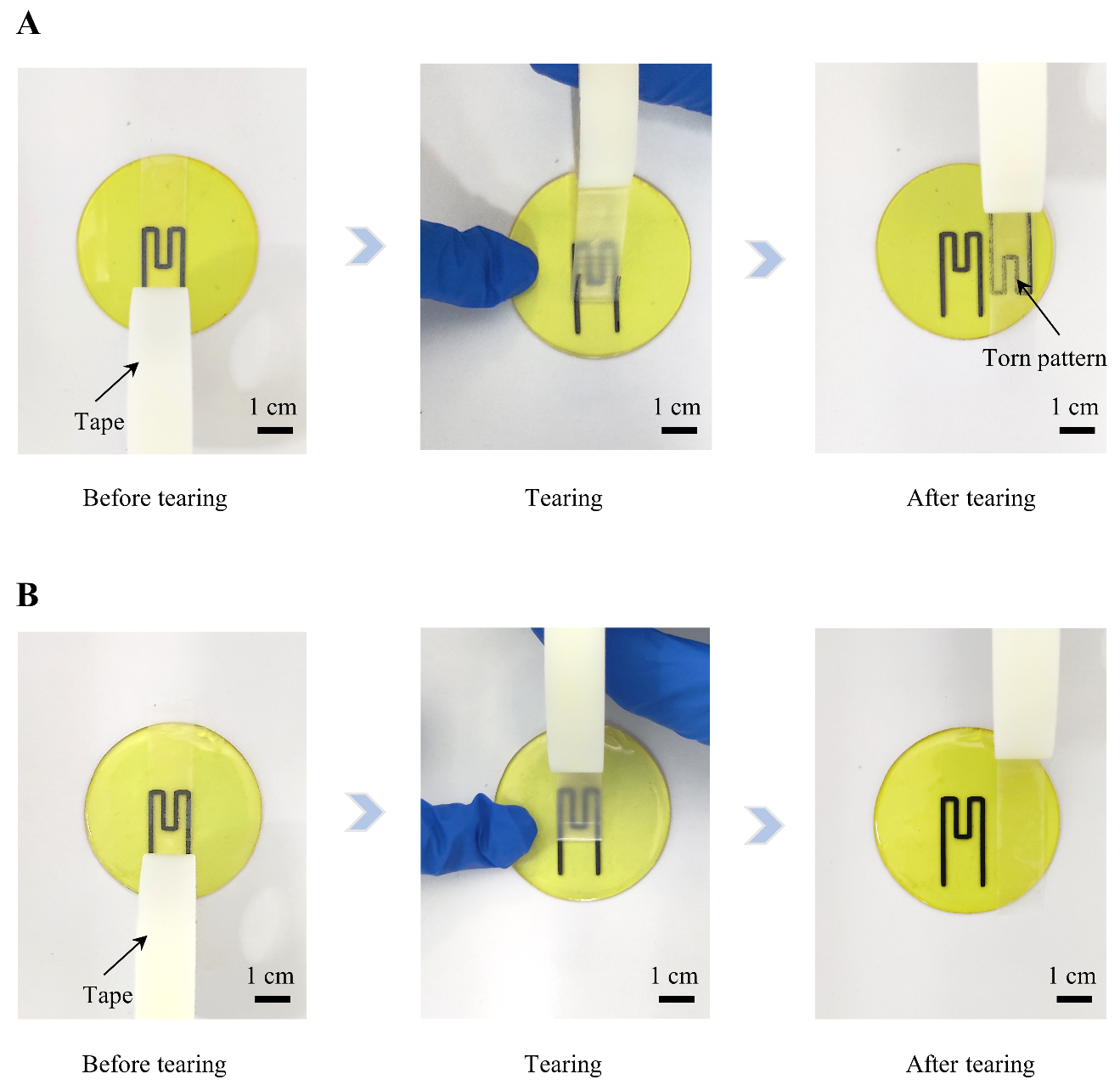


**Figure S20. Adhesion comparison test by LaserIW and surface laser-induced carbonization techniques. A,** Adhesion test of a sample by the LaserIW technique. **B,** Adhesion test of the sample by surface laser-induced carbonization.


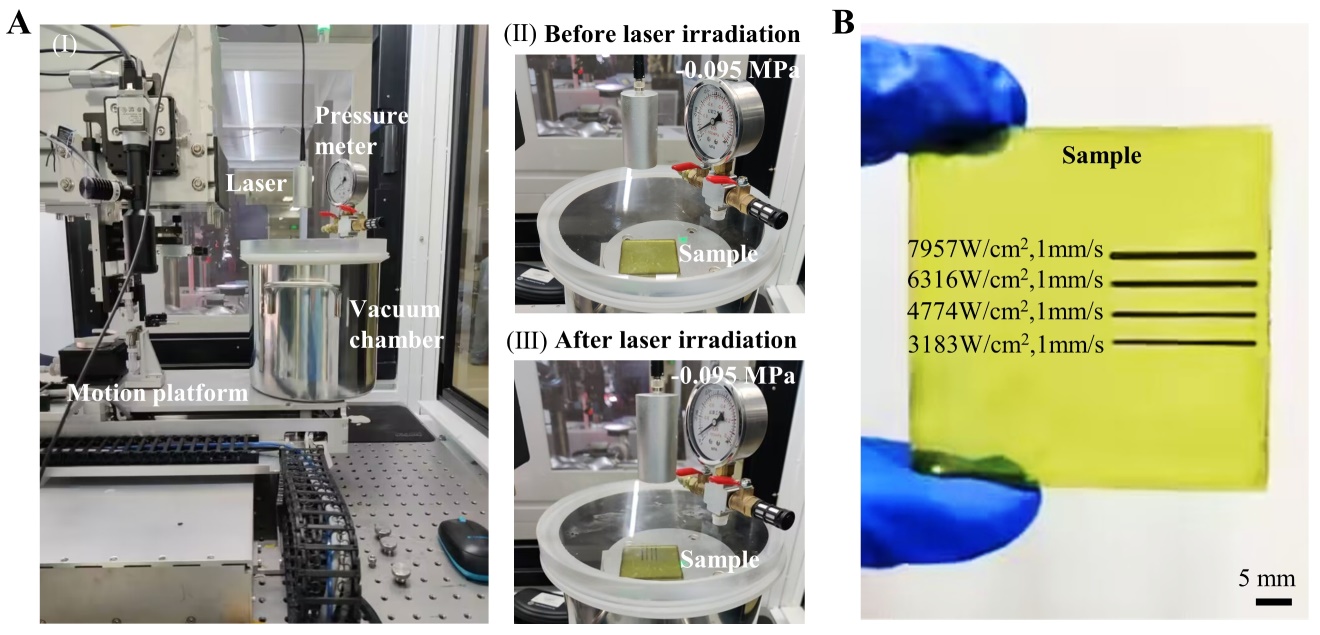


**Figure S21. Manufacturing in a vacuum environment. A,** Experimental equipment diagram includes (Ⅰ) overall photo, (Ⅱ) localized magnification before laser irradiation, and (Ⅲ) localized magnification after laser irradiation. **B,** Photo of a sample fabricated in a vacuum environment.


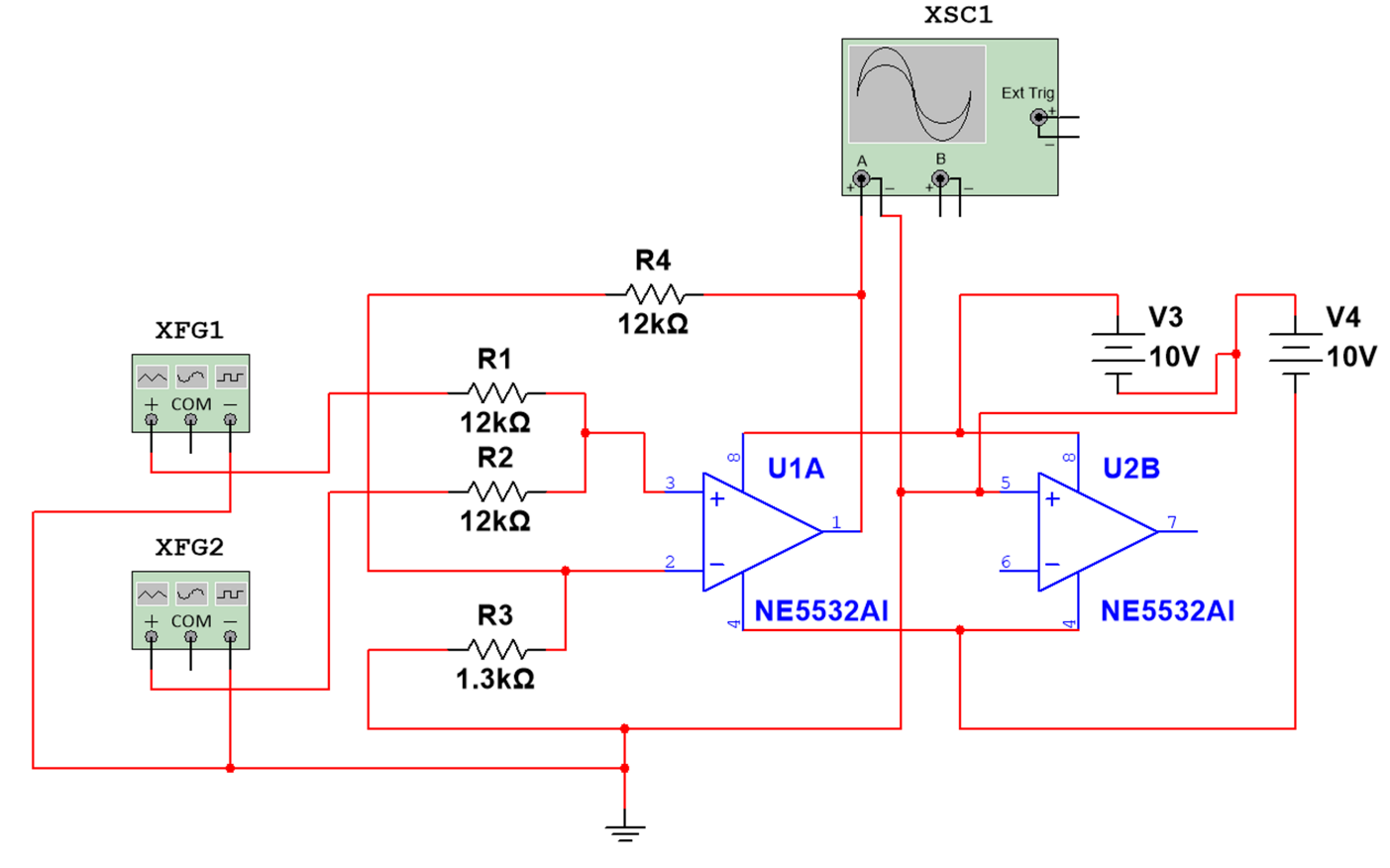


**Figure S22. Schematic diagram of the original amplifier circuit**


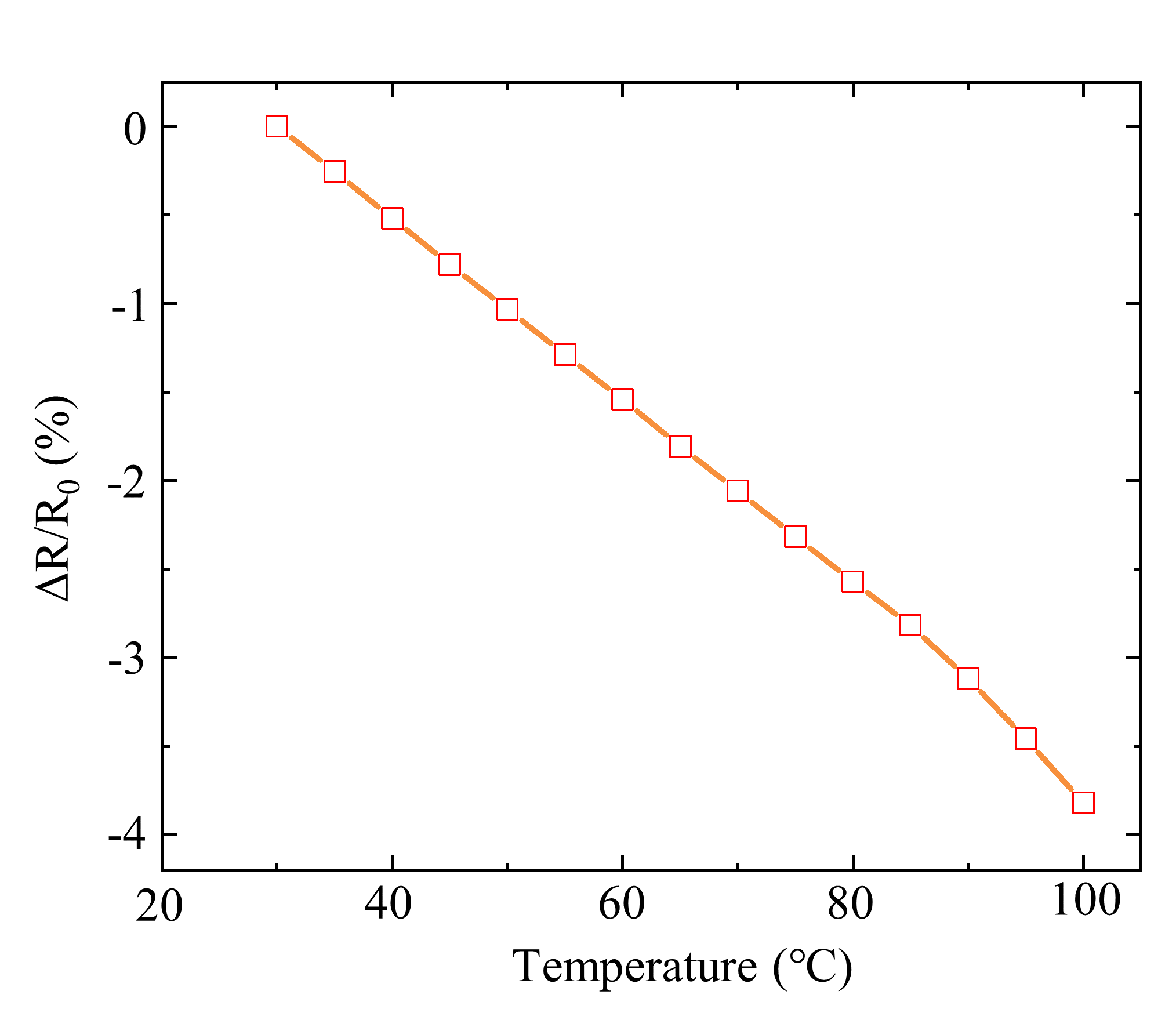


**Figure S23.** **Correlation of resistance variation rate with temperature for LIG-based resistors.**


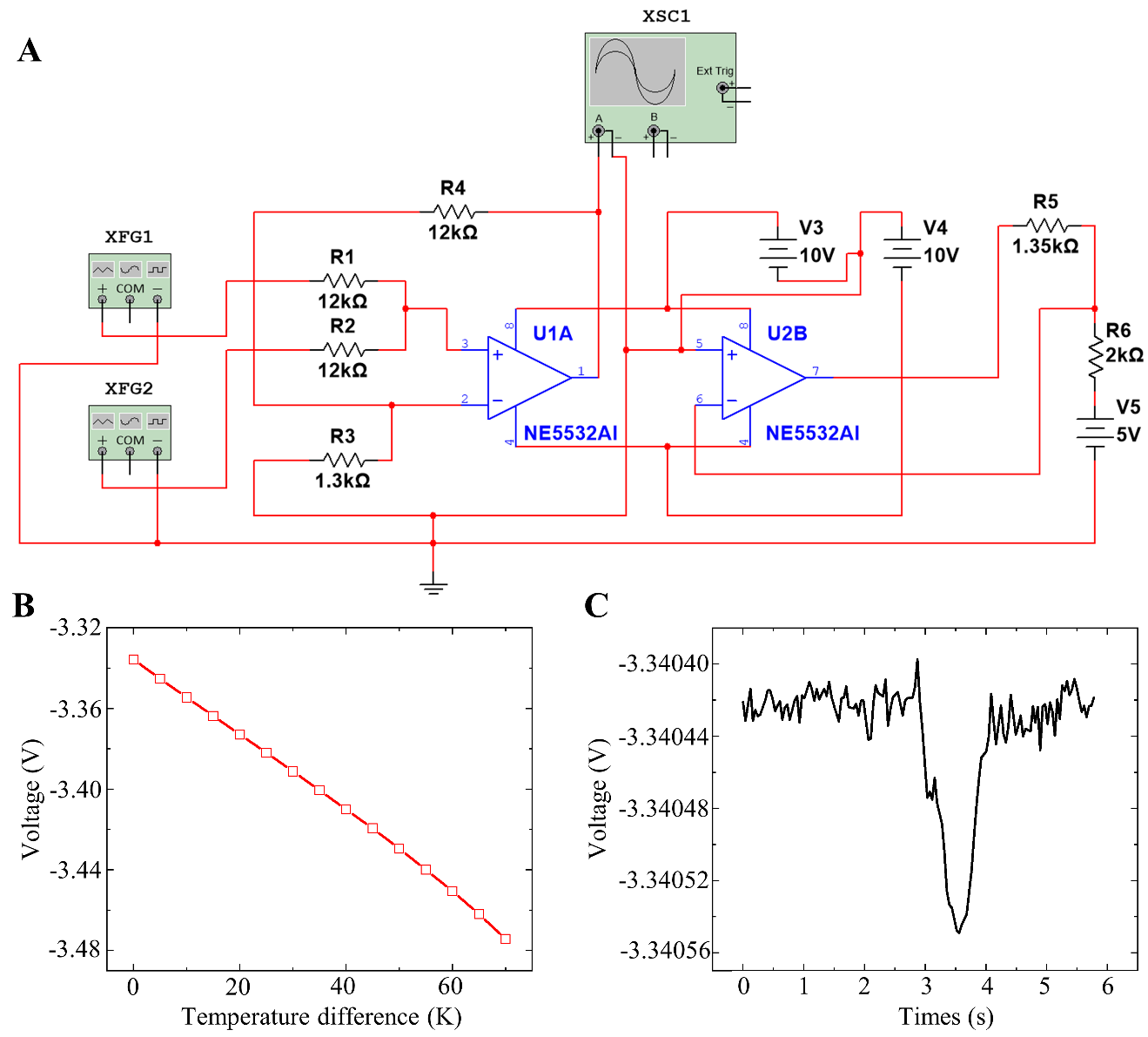


**Figure S24. Addition of temperature monitoring module to an original amplifier circuit. A,** Schematic diagram of the renovative circuit. **B,** Correlation of output voltage with temperature difference. **C,** Alteration in the output voltage as a result of the proximity of a finger to the temperature monitoring module
